# Hepatic resection alone versus in combination with pre- and post-operative transarterial chemoembolization for the treatment of hepatocellular carcinoma: A systematic review and meta-analysis

**DOI:** 10.18632/oncotarget.5426

**Published:** 2015-10-05

**Authors:** Xingshun Qi, Lei Liu, Diya Wang, Hongyu Li, Chunping Su, Xiaozhong Guo

**Affiliations:** ^1^ Department of Gastroenterology, General Hospital of Shenyang Military Area, Shenyang, China; ^2^ Xijing Hospital of Digestive Diseases, Fourth Military Medical University, Xi'an, China; ^3^ Department of Occupational and Environmental Health Sciences and the Ministry of Education Key Lab of Hazard Assessment and Control in Special Operational Environment, School of Public Health, Fourth Military Medical University, Xi'an, China; ^4^ Library of Fourth Military Medical University, Xi'an, China

**Keywords:** hepatocellular carcinoma, hepatic resection, transarterial chemoembolization, survival, recurrence

## Abstract

**Background and Aims:**

The prognosis of hepatocellular carcinoma (HCC) treated with hepatic resection may be improved by the adjunctive use of transarterial chemoembolization (TACE). This study aimed to systematically compare the outcomes between hepatic resection with and without TACE groups.

**Methods:**

All relevant randomized controlled trials (RCTs) and non-RCTs were searched by the PubMed, EMBASE, and Cochrane Library databases. Overall survival (OS) and disease-free survival (DFS) were two major outcomes. Meta-analyses were performed according to the timing of TACE (pre- or post-operative TACE). Subgroup analyses were also performed. Hazard ratios (HRs) with 95% confidence intervals (95%CIs) were calculated.

**Results:**

Overall, 55 papers were included (14 RCTs and 41 non-RCTs). Overall meta-analyses demonstrated that OS and DFS were statistically similar between hepatic resection with and without pre-operative TACE groups (HR = 1.01, 95%CI = 0.87–1.19, *P* = 0.87; HR = 0.91, 95%CI = 0.82–1.01, *P* = 0.07). Subgroup analyses of RCTs or non-RCTs showed that OS and DFS remained statistically similar between hepatic resection with and without pre-operative TACE groups. Subgroup analysis of incomplete or no tumor necrosis showed that OS was worse in hepatic resection with pre-operative TACE group than in hepatic resection without pre-operative TACE group. By contrast, subgroup analysis of complete tumor necrosis showed that DFS was better in hepatic resection with pre-operative TACE group than in hepatic resection without pre-operative TACE group.

Overall meta-analyses demonstrated that OS and DFS were better in hepatic resection with post-operative TACE group than in hepatic resection without post-operative TACE group (HR = 0.85, 95%CI = 0.72–1.00, *P* = 0.06; HR = 0.83, 95%CI = 0.73–0.94, *P* = 0.004). Subgroup analyses of RCTs, vascular invasion, or large HCC showed that OS and DFS remained better in hepatic resection with post-operative TACE group than in hepatic resection without post-operative TACE group. By contrast, subgroup analyses of non-RCTs, no vascular invasion, or small HCC showed that OS and DFS were statistically similar between the two groups.

**Conclusions:**

Post-operative TACE, rather than pre-operative TACE, may be considered as an adjunctive treatment option for HCC treated with hepatic resection.

## INTRODUCTION

Hepatocellular carcinoma (HCC) is one of the most lethal malignancies in the world [1–2]. Hepatic resection is a curative treatment option for HCC [3–4]. The current practice guidelines recommend that hepatic resection should be employed for the treatment of early HCC with single nodule and normal liver function but without clinically significant portal hypertension [5]. Recent meta-analyses have shown a statistically significant survival benefit of hepatic resection over radiofrequency ablation in small HCC, especially in HCC with >3 cm nodule [6–7]. On the other hand, accumulated evidence also suggests that the indications for hepatic resection may be further extended outside the early stage of HCC [8–10]. In clinical practices, more and more patients are considered as the candidates for hepatic resection due to the improvement of diagnostic methods, early surveillance, and surgical skills [11]. However, the residual tumor and tumor recurrence after resection remained an unresolved issue [12–13].

Transarterial chemoembolization (TACE) is recommended as the standard treatment option for HCC at intermediate stage [5]. Because the blood supply of HCC is mainly derived from hepatic artery, the use of TACE can lead to the ischemia and necrosis of tumor tissues at the embolization regions. Meta-analyses have also confirmed its significant survival benefit over no treatment [14]. Theoretically, the adjunctive use of TACE before and after hepatic resection may be effective for the prevention of tumor recurrence and improvement of survival in HCC patients. However, due to the inconsistency of conclusions among studies, the use of hepatic resection in combination with adjunctive TACE is not recommended [5].

The aim of our study was to systematically collect the relevant data comparing the outcomes of hepatic resection with and without TACE and to synthesize these data into a more unbiased and balanced result.

## RESULTS

### Study selection

**Figure 1:**
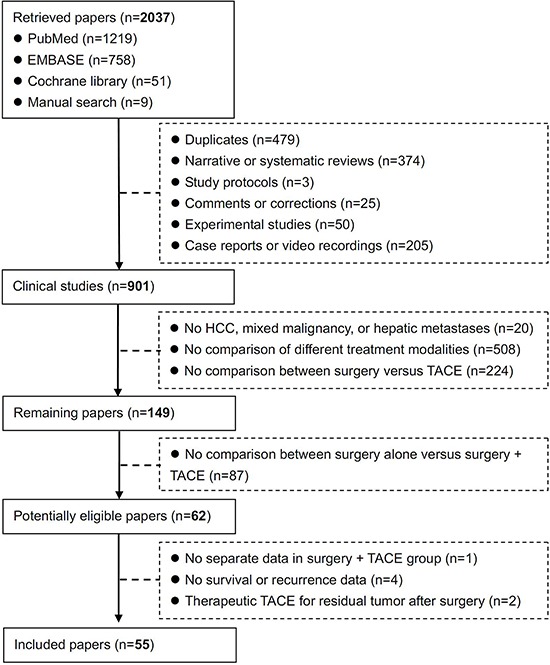
Flowchart of study inclusion

A total of 2037 papers were initially retrieved. Among them, 62 potentially eligible papers were identified. However, one paper was excluded, because the separate data in hepatic resection combined with TACE group could not be obtained [15]; four papers were excluded, because the survival and recurrence data were not provided [16–19]; and two papers were excluded, because they compared the outcomes of prophylactic versus therapeutic TACE for recurrent HCC [20–21]. Finally, 55 papers were included in the systematic review [22–76] (Figure 1).

### Study characteristics

The study characteristics were summarized in Table 1. Among them, 37 papers were cohort studies (retrospective, *n* = 25; prospective, *n* = 2; unclassified, *n* = 10), 4 papers were case-control studies (retrospective, *n* = 1; propensity score analysis, *n* = 1; unclassified, *n* = 2), and 14 papers were randomized controlled trials (RCTs). They were performed in China (*n* = 31), both China and Japan (*n* = 1), France (*n* = 2), Italy (*n* = 2), Japan (*n* = 15), and Korea (*n* = 4). The patient enrollment was initiated before and after 2000 in 36 and 19 papers, respectively. Pre-operative and post-operative TACE was performed in 32 and 22 papers, respectively. Both pre-operative and post-operative TACE were performed in one paper. As for pre-operative TACE, the interval between TACE and hepatic resection was not available in 11 papers. As for post-operative TACE, the interval between TACE and hepatic resection was not available in 4 papers.

**Table 1:** Study characteristics: an overview of included studies

First author	Journal (Year)	Region	Enrollment period	Study design	No. Pts (TACE / no TACE)	Target population	Timing of TACE	Interval between pre-operative or post-operative TACE and resection
Adachi E	Cancer (1993)	Japan, Fukuoka	1981.1–1991.6	Cohort	72 (46/26)	Patients with HCC <5 cm in maximum diameter and without portal or hepatic venous invasion and intrahepatic metastases who were thought to be at risk for disease recurrence; >3 years of follow-up after resection.	Pre-operative TACE	Average (range): 33 (5–104) days
Cheng HY	Zhonghua Zhong Liu Za Zhi (2005)	China, Shanghai	1996.6–2001.6	Retrospective cohort	1630 (987/643)	HCC who underwent hepatic resection.	Post-operative TACE	1–2 months
Cheng SQ	Zhonghua Zhong Liu Za Zhi (2004)	China, Shanghai	2000.1–2002.12	RCT	39 (23/16)	HCC who underwent hepatic resection.	Post-operative TACE	NA
Cheng SQ	Zhonghua Zhong Liu Za Zhi (2005)	China, Shanghai	2000.1–2003.1	Retrospective cohort	27 (20/7)	HCC with PVTT.	Post-operative TACE	NA
Chen XH	Zhonghua Yi Xue Za Zhi (2010)	China, Shanghai	2000.11–2007.12	Retrospective cohort	2591	HCC was divided according to tumor diameter and risk factors for residual tumor.	Post-operative TACE	1–2 months
Chen XP	Dig Surg (2007)	China, Wuhan	1990.1–2004.12	Retrospective cohort	246 (89/157)	Main tumors were centrally located (tumors confined to Couinaud's segments IV, V, and VIII).	Pre-operative TACE	Mean:26 ± 15 days
Choi GH	World J Surg (2007)	Korea, Seoul	1998.3–2005.1	Retrospective cohort	273 (120/153)	HCC who underwent curative resection.	Pre-operative TACE	Average (range): 49 (6–174) days
Di Carlo V	Hepato-gastroenterology (1998)	Italy, Milano	1989.3–1997.12	Cohort	100 (55/45)	Cirrhotic patients with HCC ≤5cm with unifocal or bifocal tumor lesions.	Pre-operative TACE	Average (range): 53 (45–140) days
Gerunda GE	Liver Transpl (2000)	Italy, Padova	1988–1997.12	Prospective cohort	37 (20/17)	HCC candidates for liver surgery.	Post-operative TACE	NA
Hanazaki K	J Am Coll Surg (2000)	Japan, Matsumoto	1983.12–1997.12	Retrospective cohort	386 (138/248); 327 (117/210)*	HCC who underwent hepatic resection.	Pre-operative TACE	NA
Harada T	Ann Surg (1996)	Japan, Fukuoka	1982.2–1994.1	Cohort	140 (105/35); 131 (98/33)*	HCC who underwent hepatic resection.	Pre-operative TACE	Average (range): 72.9 ± 52.0 (21–327) days
Izumi R	Hepatology (1994)	Japan, Kanazawa	1987.1–1992.8	RCT	50 (23/27)	Patients with invasive HCC with vascular invasion and/or intrahepatic metastasis, who underwent curative hepatic resection in which all the macroscopic HCC was removed during surgery.	Post-operative TACE	Mean (range): 38.4 days (21–84)
Jianyong L	Ann Hepatol (2014)	China, Chengdu	2005.6–2008.8	Retrospective cohort	656 (183/405)	HCC who underwent curative resection.	Pre-operative TACE	Mean: 135 days
Kaibori M	Anticancer Research (2006)	Japan, Osaka	1992.2–2005.2	Cohort	245 (115/128)	HCC who underwent curative hepatectomy was divided according to the preoperative ICGR15.	Pre-operative TACE	NA
Kaibori M	Dig Dis Sci (2012)	Japan, Osaka	2004.1–2007.6	RCT	124 (81/43)	HCC who underwent curative resection.	Pre-operative TACE	Mean: 21.2 ± 10.8 days in selective TACE group; 23.0 ± 13.2 days in whole-liver chemolipiodolization group
Kang JY	Korean J Hepatol (2010)	Korea, Seoul	1997.1–2007.12	Case-control	96 (32/64)	HCC who underwent hepatic resection.	Pre-operative TACE	Mean:102.9 ± 130.7 days
Kim IS	Aliment Pharmacol Ther (2008)	Korea, Seoul	1995.1–2000.12	Retrospective cohort	334 (97/237)	HCC who underwent curative resection.	Pre-operative TACE	Median (range):4 weeks (1–16)
Kishi Y	Hepato-gastroenterology (2012)	Japan, Tokyo	1994.2–2008.10	Cohort	227 (69/158)	HCC who underwent curative resection.	Pre-operative TACE	Within 3 months
Lee KT	J Surg Oncol (2009)	China, Taiwan	2000.1–2006.6	Retrospective cohort	350 (114/236)	HCC who underwent hepatic resection.	Post-operative TACE	Median (range):66.5 ± 110.9 days (2–286)
Li F	Ir J Med Sci (2014)	China, Tianjin	2006.2–2009.5	Retrospective cohort	60 (26/34)	HBV-related HCC, no prior treatments for HCC except hepatic curative resection treatment, BCLC stage B, Child-Pugh stage A or B, HBsAg positive, no distant metastasis, and no contraindication for laparotomy.	Post-operative TACE	1 month
Li JQ	J Cancer Res Clin Oncol (1995)	China, Guangzhou	1990.4–1993.12	RCT	140 (70/70)	HCC patients undergoing radical (*n* = 94) and palliative resection (*n* = 46).	Post-operative TACE	3–4 weeks
Li KW	Hepato-gastroenterology (2012)	China, Chengdu	2005.7–2010.8	Retrospective cohort	76 (35/41)	HCC, no lymph node involvement and distant metastasis, no macroscopic venous invasion.	Post-operative TACE	4–6 weeks
Li Q	Dig Surg (2006)	China, Tianjin	1998.1–2001.1	RCT	84 (39/45)	HCC, no previous management, solitary or multiple tumors mainly located in one lobe of the liver, no distant metastases, Child-Pugh stage A or B.	Post-operative TACE	4 weeks
Li Q	World J Surg (2006)	China, Tianjin	1998.1–2001.1	RCT	72 (35/37)	HCC, no previous management, tumor thrombus in the first branch and/or main trunk of the portal vein, solitary or multiple tumors mainly located in one lobe of the liver, no distant metastasis, Child-Pugh stage A or B, no contraindication for laparotomy.	Post-operative TACE	4 weeks
Liu YJ	Zhonghua Fang She Xue Za Zhi (2010)	China, Beijing	1997.1–2009.12	Retrospective cohort	386 (230/156)	HCC who underwent hepatic resection.	Pre-and/or post-operative TACE	Pre-operative TACE: Median (range): 7 weeks (l-34); Mean: 7 ± 3 Post-operative TACE: Median (range): 8 weeks (2–124); Mean: 9 ± 2
Lu CD	World J Surg (1999)	China, Hangzhou; Japan, Fukui	1988–1994	Retrospective cohort	120 (44/76)	HCC patients excluding tumor diameter ≤2 cm, hepatectomy-related mortality, portal vein or hepatic vein tumor thrombus identified by imaging or operation, and lymph node or extrahepatic metastasis.	Pre-operative TACE	Tumors 2–8 cm in diameter: 29 ± 10 days (range: 9–42 days); Tumors >8 cm in diameter: 8 ± 75 days (range: 30–312 days)
Majno PE	Ann Surg (1997)	France, Villejuif	1985.1–1995.12	Retrospective cohort	76 (49/27)	Histologically confirmed HCC associated with cirrhosis who underwent liver resection.	Pre-operative TACE	NA
Nagasue N	Surgery (1989)	Japan, Hiroshima	1980.1–1987.12	Retrospective cohort	138 (31/107)	Primary HCC who underwent liver resection with total extirpation of tumor.	Pre-operative TACE	Mean:130 days
Nishikawa H	Int J Oncol (2013)	Japan, Osaka	2004.1–2012.6	Retrospective cohort	235 (110/125)	Treatment-naïve HCC who underwent hepatic resection.	Pre-operative TACE	NA
Ochiai T	Hepato-gastroenterology (2003)	Japan, Shiga	1978.10–1994.4	Retrospective cohort	148 (100/48)	Solitary HCC who underwent hepatic resection.	Pre-operative TACE	Average:35.7 days
Park JH	Cardiovasc Intervent Radiol (1993)	Korea, Seoul	1987.2–1991.9	Retrospective cohort	65 (45/20)	Patients who developed recurrent HCC after resection underwent TACE between February 1987 and September 1991.	Pre-operative TACE	NA
Paye F	Arch Surg (1998)	France, Clichy	1986.12–1992.1	Case-control	48 (24/24)	HCC who underwent resection.	Pre-operative TACE	Mean:45 ± 40 days
Peng BG	Am J Surg (2009)	China, Guangzhou	1996.1–2006.12	RCT	104 (51/53)^$^	HCC with PVTT.	Post-operative TACE	NA
Ren ZG	World J Gastroenterol (2004)	China, Shanghai	1995.1–1998.12	Retrospective case-control	549 (185/364)	All HCC lesions were removed, no lymph node involvement, no distant metastasis.	Post-operative TACE	2 months
Sasaki A	Eur J Surg Oncol (2006)	Japan, Beppu	1982.7–2003.4	Retrospective cohort	239 (109/126)	HCC who underwent curative resection.	Pre-operative TACE	Mean: 33.1 days; Median (range): 18.0 days (2–276)
Shi HY	J Surg Oncol (2014)	China, Taiwan	1996–2009	Propensity score analysis	1296 (648/648)	HCC who underwent liver resection.	Pre-operative TACE	NA
Sugo H	World J Surg (2003)	Japan, Tokyo	1979.9–2000.3	Retrospective cohort	227 (146/81)	HCC who underwent liver resection.	Pre-operative TACE	NA
Tang QH	Academic J Second Military Medical University (2009)	China, Shanghai	2001.7–2003.12	RCT	108 (52/56)	Resectable large HCC (≥5 cm).	Pre-operative TACE	5–8 weeks
Uchida M	World J Surg (1996)	Japan, Izumo	1986.4–1991.11	Retrospective cohort	128 (60/68)	HCC who underwent curative resection of the tumor.	Pre-operative TACE	Mean:42.3 ± 28.4 days
Wang QX	Zhonghua Wai Ke Za Zhi (2009)	China, Shanghai	2004.1–2007.6	Retrospective cohort	260 (104/156)	HCC who underwent curative resection.	Post-operative TACE	Mean:1.7 months (1–6)
Wang TH	Chinese J Cancer Prevention and Treatment (2010)	China, Shanghai	1997.1–2007.12	Retrospective cohort	176 (51/125)	Huge HCC who had undergone surgical resection.	Pre-operative TACE	Mean:60 days (18–135)
Wu CC	Br J Surg (1995)	China, Taiwan	1983.1–1991.12	RCT	52 (24/28); 49 (23/26)^#^	Resectable large HCC.	Pre-operative TACE	Mean:1.8±0.6 weeks (range: 1.0–3.0)
Xiao EH	Zhonghua Zhong Liu Za Zhi (2005)	China, Changsha	1992.2–1999.2	Cohort	139 (81/58)	Histologically confirmed HCC who underwent liver resection.	Pre-operative TACE	NA
Xiao YP	World Chinese J Digestology (2012)	China, Guangxi	2005.10–2010.10	Retrospective cohort	120 (88/32)	HCC who underwent curative resection; with high-risk factor of recurrence: 1) vascular tumor thrombus; 2) multiple nodules; 3) pre-operative AFP >200 μg/L; 4) large HCC (diameter >5 cm).	Post-operative TACE	3–6 weeks
Xi T	Hepato-gastroenterology (2012)	China, Shanghai	1996.2–2001.9	Cohort	721 (145/576)	HCC who underwent R0 partial hepatectomy.	Post-operative TACE	4–9 weeks
Xu F	Academic J Second Military Medical University (2012)	China, Shanghai	2008.9–2009.12	RCT	117 (59/58)	Small HCC (diameter ≤5 cm), single nodule, no vascular invasion, no residual tumor, no extrahepatic metastasis, Child-Pugh score ≤7.	Post-operative TACE	1 month
Xu F	Academic J Second Military Medical University (2012)	China, Shanghai	2008.1–2008.12	Prospective cohort	104 (56/48)	HCC who under curative resection; number of tumor < 4; no extrahepatic spread; no residual tumor.	Post-operative TACE	1 month
Yamasaki S	Jpn J Cancer Res (1996)	Japan, Tokyo	1987.7–1989.12	RCT	115 (57/58)	Untreated non-recurrent HCC; age ≤65.	Pre-operative TACE	NA
Yanaga K	HPB (2014)	Japan	2000.1–2013.10	Cohort	213 (37/176)	HCC who underwent liver resection.	Pre-operative TACE	NA
Yang PS	Liver Transpl (2010)	China, Taiwan	1995.5–2009.6	Cohort	241 (206/35)	HCC with Milan criteria who underwent liver resection.	Pre-operative TACE	NA
Yan Q	Chin Med J (2013)	China, Hangzhou	2005.1–2008.6	Cohort	44 (19/25)	HBV-related HCC who underwent local and regional liver resection, single tumor < 8 cm; ≤3 tumor < 3 cm; no residual tumor; no distant metastasis.	Post-operative TACE	2 months
Yu ZP	J Pract Med (2009)	China, Wenzhou	2000.1–2005.6	RCT	97 (50/47)	HCC who underwent curative resection.	Post-operative TACE	Within 2 weeks (n=36); beyond 2 weeks (n=14)
Zhang Z	Cancer (2000)	China, Shanghai	1990.1–1995.12	Retrospective cohort	1457 (1275/182)	Resectable HCC.	Pre-operative TACE	From 15 days to 8 months
Zhong C	J Cancer Res Clin Oncol (2009)	China, Guangzhou	2001.1–2004.3	RCT	115 (57/58)	Stage IIIA HCC (multiple tumors >5 cm or tumor involving a major branch of the portal or hepatic vein(s) (UICC TNM staging system, sixth edition)).	Post-operative TACE	4-6 weeks
Zhou WP	Ann Surg (2009)	China, Shanghai	2001.7–2003.12	RCT	108 (52/56)	Resectable large HCC (≥5 cm).	Pre-operative TACE	5-8 weeks

***Abbreviations:*** HCC, hepatocellular carcinoma; NA, not available; PVTT, portal vein tumor thrombus; RCT, randomized controlled trial; TACE, transarterial chemoembolization.

***Notes:***

* patients who were finally included in the disease-free survival analysis.

$ patients lost to follow-up were excluded from the survival analysis.

# patients who were finally included in the survival analysis.

### Study quality

Of the 37 cohort studies, 7 had 0–3 points, 27 had 4–6 points, and 3 had 7–8 points (Supplementary Table 1). All of the 4 case-control studies had 4–6 points (Supplementary Table 2). Of the 14 RCTs, 1, 8, and 5 had high, unclear, and low risk of bias in random sequence generation, respectively; 2, 8, and 4 had high, unclear, and low risk of bias in allocation concealment, respectively; 3 and 11 had high and unclear risk of bias in blinding of participants and personnel, respectively; 13 and 1 had unclear and low risk of bias in blinding of outcome assessment, respectively; 1, 8, and 5 had high, unclear, and low risk of bias in incomplete outcome data addressed, respectively; and 1 and 13 had high and low risk of bias in selective reporting, respectively (Supplementary Table 3).

### Pre-operative TACE

#### Overall survival (OS)

**Figure 2:**
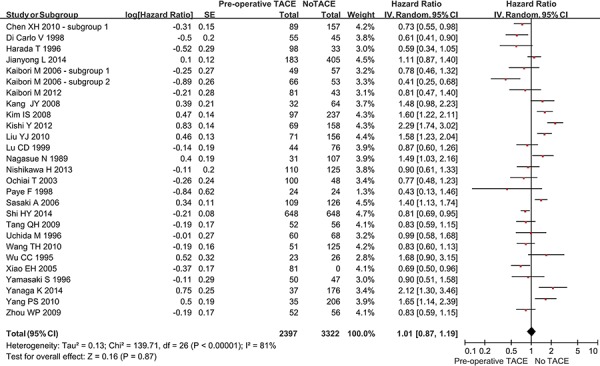
Forest plots comparing the overall survival between hepatic resection with and without pre-operative TACE groups

Twenty-four studies reported the OS rate in the two groups. The overall meta-analysis demonstrated a statistically similar OS between hepatic resection with and without pre-operative TACE groups (hazard ratio [HR] = 1.01, 95% confidence interval [CI] = 0.87–1.19, *P* = 0.87) (Figure 2). The heterogeneity among studies was statistically significant (*P* < 0.00001; I^2^ = 81%). The funnel plot suggested a proof of publication bias (Supplementary Figure 1).

In the subgroup analysis of complete tumor necrosis after TACE, the OS remained statistically similar between the two groups (HR = 1.02, 95%CI = 0.63–1.66, *P* = 0.93) (Supplementary Figure 2). However, in the subgroup analysis of incomplete or no tumor necrosis after TACE, the OS was statistically significantly worse in hepatic resection with pre-operative TACE group than in hepatic resection without pre-operative TACE group (HR = 2.01, 95%CI = 1.22–3.31, *P* = 0.006). The subgroup difference was statistically significant (*P* = 0.06; I^2^ = 72.2%).

Regardless of large or small HCC, the OS remained statistically similar between the two groups (in large HCC: HR = 0.85, 95%CI = 0.68–1.07, *P* = 0.18; in small HCC: HR = 1.10, 95%CI = 0.58–2.07, *P* = 0.77) (Supplementary Figure 3). There was no statistically significant subgroup difference (*P* = 0.46; I^2^ = 0%).

In the subgroup analysis of cirrhotic patients, the OS was statistically significantly better in hepatic resection with pre-operative TACE group than in hepatic resection without pre-operative TACE group (HR = 0.67, 95%CI = 0.47–0.96, *P* = 0.03) (Supplementary Figure 4). By comparison, in the subgroup analysis of non-cirrhotic patients, the OS was statistically similar between the two groups (HR = 0.63, 95% CI = 0.17–2.32, *P* = 0.48). There was no statistically significant subgroup difference (*P* = 0.92; I^2^ = 0%).

Regardless of randomized or non-randomized studies, the OS remained statistically similar between the two groups (in RCT: HR = 0.90, 95%CI = 0.73–1.10, *P* = 0.30; in non-RCT: HR = 1.03, 95%CI = 0.86–1.23, *P* = 0.77) (Supplementary Figure 5). There was no statistically significant subgroup difference (*P* = 0.33; I^2^ = 0%).

#### Disease-free survival (DFS)

**Figure 3:**
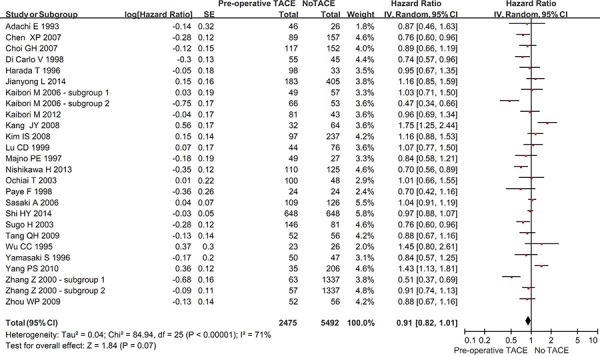
Forest plots comparing the disease-free survival between hepatic resection with and without pre-operative TACE groups

Twenty-four studies reported the DFS rate in the two groups. The overall meta-analysis demonstrated a better DFS in hepatic resection with pre-operative TACE group than in hepatic resection without pre-operative TACE group. But the difference was not statistically significant (HR = 0.91, 95% CI = 0.82–1.01, *P* = 0.07) (Figure 3). The heterogeneity among studies was statistically significant (*P* < 0.00001; I^2^ = 71%). The funnel plot suggested a proof of publication bias (Supplementary Figure 6).

In the subgroup analysis of complete tumor necrosis after TACE, the DFS was statistically significantly better in hepatic resection with pre-operative TACE group than in hepatic resection without pre-operative TACE group (HR = 0.67, 95% CI = 0.58–0.77, *P* < 0.00001) (Supplementary Figure 7). However, in the subgroup analysis of incomplete or no tumor necrosis after TACE, the DFS was statistically similar between the two groups (HR = 1.13, 95% CI = 0.94–1.35, *P* = 0.20). The subgroup difference was statistically significant (*P* < 0.00001; I^2^ = 95.1%).

Regardless of large or small HCC, the DFS was statistically similar between the two groups (in large HCC: HR = 0.86, 95% CI = 0.69–1.06, *P* = 0.15; in small HCC: HR = 1.10, 95% CI = 0.80–1.50, *P* = 0.56) (Supplementary Figure 8). There was no statistically significant subgroup difference (*P* = 0.20; I^2^ = 39.8%).

In the subgroup analysis of cirrhotic patients, the DFS was statistically significantly better in hepatic resection with pre-operative TACE group than in hepatic resection without pre-operative TACE group (HR = 0.77, 95% CI = 0.62–0.95, *P* = 0.01) (Supplementary Figure 9). No study was identified in the subgroup analysis of non-cirrhotic patients.

Regardless of randomized or non-randomized studies, the DFS was statistically similar between the two groups (in RCT: HR = 0.92, 95% CI = 0.79–1.07, *P* = 0.26; in non-RCT: HR = 0.90, 95% CI = 0.81–1.02, *P* = 0.09) (Supplementary Figure 10). There was no statistically significant subgroup difference (*P* = 0.88; I^2^ = 0%).

#### Free of recurrence

Two studies reported the recurrence rate in the two groups. Both of them demonstrated a higher overall rate free of recurrence in hepatic resection without pre-operative TACE group than in hepatic resection with pre-operative TACE group (39.7% versus 27.5%; 62% versus 51%).

### Post-operative TACE

#### OS

**Figure 4:**
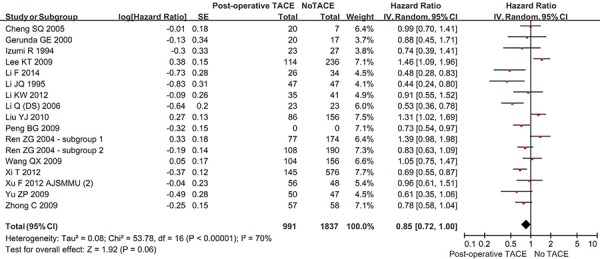
Forest plots comparing the overall survival between hepatic resection with and without post-operative TACE groups

Sixteen studies reported the OS rate in the two groups. The overall meta-analysis demonstrated a better OS in hepatic resection with post-operative TACE group than in hepatic resection without post-operative TACE group. But the difference was not statistically significant (HR = 0.85, 95% CI = 0.72–1.00, *P* = 0.06) (Figure 4). The heterogeneity among studies was statistically significant (*P* < 0.00001; I^2^ = 70%). The funnel plot suggested a proof of publication bias (Supplementary Figure 11).

In the subgroup analysis of vascular invasion, the OS was statistically significantly better in hepatic resection with post-operative TACE group than in hepatic resection without post-operative TACE group (HR = 0.80, 95% CI = 0.69–0.92, *P* = 0.002) (Supplementary Figure 12). However, in the subgroup analysis of no vascular invasion, the OS was statistically similar between the two groups (HR = 0.90, 95% CI = 0.59–1.38, *P* = 0.64). The subgroup difference was not statistically significant (*P* = 0.57; I^2^ = 0%).

In the subgroup analysis of large HCC, the OS was statistically significantly better in hepatic resection with post-operative TACE group than in hepatic resection without post-operative TACE group (HR = 0.77, 95% CI = 0.65–0.90, *P* = 0.001) (Supplementary Figure 13). In the subgroup analysis of small HCC, the OS was better in hepatic resection with post-operative TACE group than in hepatic resection without post-operative TACE group. But the difference was not statistically significant (HR = 1.39, 95%CI = 0.98–1.98, *P* = 0.07). The subgroup difference was statistically significant (*P* = 0.003; I^2^ = 89.0%).

In the subgroup analysis of randomized studies, the OS was statistically significantly better in hepatic resection with post-operative TACE group than in hepatic resection without post-operative TACE group (HR = 0.67, 95% CI = 0.57–0.79, *P* < 0.00001) (Supplementary Figure 14). However, in the subgroup analysis of non-randomized studies, the OS was statistically similar between the two groups (HR = 0.98, 95% CI = 0.81–1.19, *P* = 0.82). The subgroup difference was statistically significant (*P* = 0.003; I^2^ = 88.7%).

#### DFS

**Figure 5:**
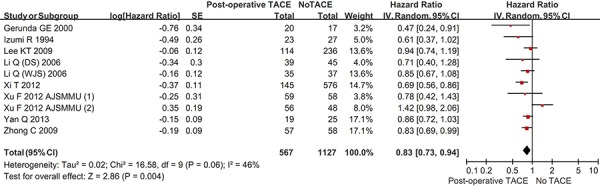
Forest plots comparing the disease-free survival between hepatic resection with and without post-operative TACE groups

Ten studies reported the DFS rate in the two groups. The overall meta-analysis demonstrated a statistically significantly better DFS in hepatic resection with post-operative TACE group than in hepatic resection without post-operative TACE group (HR = 0.83, 95% CI = 0.73–0.94, *P* = 0.004) (Figure 5). The heterogeneity among studies was statistically significant (*P* = 0.06; I^2^ = 46%). The funnel plot suggested a proof of publication bias (Supplementary Figure 15).

In the subgroup analysis of vascular invasion, the DFS was statistically significantly better in hepatic resection with post-operative TACE group than in hepatic resection without post-operative TACE group (HR = 0.82, 95% CI = 0.71–0.94, *P* = 0.004) (Supplementary Figure 16). However, in the subgroup analysis of no vascular invasion, the DFS was statistically similar between the two groups (HR = 0.97, 95%CI = 0.60–1.56, *P* = 0.90). The subgroup difference was not statistically significant (*P* = 0.50; I^2^ = 0%).

In the subgroup analysis of large HCC, the DFS was statistically significantly better in hepatic resection with post-operative TACE group than in hepatic resection without post-operative TACE group (HR = 0.80, 95% CI = 0.69–0.92, *P* = 0.003) (Supplementary Figure 17). In the subgroup analysis of small HCC, the DFS was better in hepatic resection with post-operative TACE group than in hepatic resection without post-operative TACE group. But the difference was not statistically significant (HR = 0.85, 95% CI = 0.72–1.01, *P* = 0.07). The subgroup difference was not statistically significant (*P* = 0.54; I^2^ = 0%).

In the subgroup analysis of randomized studies, the DFS was statistically significantly better in hepatic resection with post-operative TACE group than in hepatic resection without post-operative TACE group (HR = 0.81, 95%CI = 0.71–0.92, *P* = 0.001) (Supplementary Figure 18). However, in the subgroup analysis of non-randomized studies, the DFS was statistically similar between the two groups (HR = 0.86, 95%CI = 0.68–1.09, *P* = 0.21). The subgroup difference was statistically significant (*P* = 0.66; I^2^ = 0%).

#### Free of recurrence

**Figure 6:**
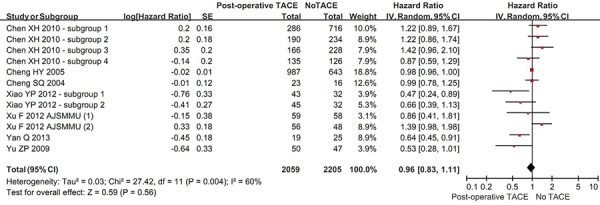
Forest plots comparing the rate of being free of recurrence between hepatic resection with and without post-operative TACE groups

Eight studies reported the recurrence rate in the two groups. The overall meta-analysis demonstrated a statistically similar rate free of recurrence between hepatic resection with and without post-operative TACE groups (HR = 0.96, 95% CI = 0.83–1.11, *P* = 0.56) (Figure 6). The heterogeneity among studies was statistically significant (*P* = 0.004; I^2^ = 60%). The funnel plot suggested a proof of publication bias (Supplementary Figure 19).

In the subgroup analysis of vascular invasion, the rate free of recurrence was statistically significantly higher in hepatic resection with post-operative TACE group than in hepatic resection without post-operative TACE group (HR = 0.58, 95% CI = 0.38–0.87, *P* = 0.008) (Supplementary Figure 20). However, in the subgroup analysis of no vascular invasion, the rate free of recurrence was statistically similar between the two groups (HR = 0.92, 95% CI = 0.53–1.61, *P* = 0.77). The subgroup difference was not statistically significant (*P* = 0.19; I^2^ = 42.7%).

Regardless of large or small HCC, the rate free of recurrence was statistically similar between the two groups (in large HCC: HR = 0.82, 95% CI = 0.53–1.28, *P* = 0.39; in small HCC: HR = 0.97, 95% CI = 0.68–1.38, *P* = 0.86) (Supplementary Figure 21). There was no statistically significant subgroup difference (*P* = 0.58; I^2^ = 0%).

Regardless of randomized or non-randomized studies, the rate free of recurrence was statistically similar between the two groups (in RCT: HR = 0.84, 95% CI = 0.58–1.21, *P* = 0.34; in non-RCT: HR = 0.98, 95% CI = 0.82–1.17, *P* = 0.83) (Supplementary Figure 22). There was no statistically significant subgroup difference (*P* = 0.44; I^2^ = 0%).

## DISCUSSION

We identified a relatively large number of relevant papers evaluating the role of adjunctive TACE for the management of HCC patients treated with hepatic resection. The main findings of our systematic review and meta-analysis were as follows: 1) pre-operative TACE did not significantly improve the OS and DFS of HCC patients treated with hepatic resection; and 2) post-operative TACE significantly improved the DFS of HCC patients treated with hepatic resection, but not the OS or recurrence. Before our findings were discussed, several previous meta-analyses should be acknowledged.

As for the pre-operative TACE, 3 meta-analyses were reported. In 2011, Wang et al. published a meta-analysis of 3 RCTs involving 257 patients to identify the effect of pre-operative TACE for resectable HCC [77]. They suggested no significant benefits of pre-operative TACE for the 5-year DFS and OS. In 2013, Yu et al. performed a meta-analysis of 7 retrospective studies to evaluate the effect of pre-operative TACE on resectable HCC [78]. There was a trend toward a better 3-year DFS in pre-operative TACE group, but the difference was not statistically significant. By comparison, the 5-year DFS rate was significantly higher in pre-operative TACE group than in non-TACE group. In addition, the 5-year OS rate was significantly improved by pre-operative TACE. At the same year, Zhou et al. also conducted a larger meta-analysis of 21 studies (4 RCTs and 17 vnon-RCTs) involving 3210 HCC patients to explore the benefits of pre-operative TACE for resectable HCC [79]. They demonstrated that pre-operative TACE did not significantly improve the DFS or OS of resectable HCC.

As for the post-operative TACE, 1 meta-analysis was reported. In 2010, Zhong et al. performed a meta-analysis of 6 RCTs involving 659 HCC patients to evaluate the efficacy of post-operative TACE [80]. They found that post-operative TACE significantly decreased the 1- and 3-year mortality of HCC with multiple nodules of >5 cm or vascular invasion.

Furthermore, 2 papers evaluated both pre-operative and post-operative TACE in combination with hepatic resection for HCC. In 2003, Mathurin et al. published a meta-analysis to evaluate the adjunctive chemotherapy after curative resection for HCC [81]. In their meta-analysis, the modality of chemotherapy was not restricted. Both transarterial chemotherapy with and without embolization were included. Among them, 10 and 7 studies evaluated the roles of pre- and post-operative transarterial chemotherapy, respectively. They found that only post-operative transarterial chemotherapy, but not pre-operative transarterial chemotherapy, improved the survival and decreased the probability of no recurrence. In 2014, Cheng et al. performed a meta-analysis of 10 RCTs involving 909 patients to assess the beneficial and harmful effects of pre-operative and post-operative TACE for curative resection of HCC [82]. Among them, 4 and 6 trials assessed the outcomes of pre-operative and post-operative TACE, respectively. In line with the findings by Mathurin et al., they also found that pre-operative TACE did not improve DFS and OS for curative resection of HCC, but post-operative TACE achieved significant improvement of DFS and OS in patients with tumor size >5cm.

Most of previous meta-analyses suggested that the adjunctive use of pre-operative TACE was not effective, but post-operative TACE might be beneficial for the improvement of DFS and OS. By comparison, our study had several strengths. First, both pre-operative and post-operative TACE were evaluated. Second, both RCTs and non-RCTs were included. Certainly, the subgroup analysis was divided into RCTs and non-RCTs groups. In addition, non-RCTs could reflect the real-world conditions more accurately. Third, both OS and DFS were evaluated. Fourth, the search strategy was more extensive, and the number of included studies was larger. Fifth, the study quality was strictly evaluated according to the well-known scales or tools. Sixth, the subgroup analysis was performed to explore the efficacy of pre-operative and post-operative TACE. Seventh, the HR was calculated to show a trend over time, but not an odds ratio at some time point.

In agreement with the recommendations from current practice guidelines [5], the overall meta-analysis did not find any significant benefits of TACE before hepatic resection. Notably, if the tumor necrosis was incomplete or lacking, pre-operative TACE would deteriorate the OS of HCC patients treated with hepatic resection. By comparison, if the tumor necrosis was complete, pre-operative TACE would improve the DFS of HCC patients treated with hepatic resection. Thus, whether or not the use of TACE was valuable before hepatic resection might be largely dependent upon the grade of tumor necrosis. Further studies should be helpful to identify the candidates who had a higher probability of complete tumor necrosis induced by pre-operative TACE.

We also found that the efficacy of pre-operative TACE was not influenced by the tumor size or study design. Additionally, in the setting of liver cirrhosis, pre-operative TACE might significantly improve the OS and DFS of HCC patients treated with hepatic resection. However, it should be noted that only a small number of studies were included in these subgroup analyses.

In contrast with the previous meta-analyses [81–82], we did not find a statistically significantly better OS in post-operative TACE group than in no post-operative TACE group. However, a trend towards the improvement of OS by post-operative TACE should be clearly recognized. Besides, the DFS was significantly improved by post-operative TACE. As we looked at the subgroup results, the OS and DFS benefit of post-operative TACE was statistically significant in patients with more advanced HCC (i.e., vascular invasion or large HCC). On the contrary, the OS and DFS were statistically similar between the two groups in patients with less advanced HCC (i.e., no vascular invasion and small HCC). Indeed, both vascular invasion and large HCC are associated with a higher rate of tumor recurrence after hepatic resection. In such patients, the adjunctive use of post-operative TACE might be more worthwhile to further improve the patients' prognosis.

We also found that the subgroup results of post-operative TACE were different between RCTs and non-RCTs. In the subgroup meta-analysis of RCTs, the OS and DFS were significantly improved by post-operative TACE. More importantly, we did not observe any statistically significant heterogeneity among these included RCTs (I^2^ = 0% in both subgroup analyses). By contrast, in the subgroup meta-analyses of non-RCTs, the OS and DFS were not significantly different between hepatic resection with and without post-operative TACE groups. The heterogeneity among studies was significant (I^2^ = 69% in the OS analysis; I^2^ = 73% in the DFS analysis). This discrepancy may be attributed to the potential bias in the patient and treatment selection among the non-RCTs, in which post-operative TACE might be more frequently employed for the patients with a higher probability of tumor recurrence after hepatic resection. Thus, the outcomes became similar between the two groups.

Our study had several limitations. First, we observed statistically significant heterogeneities in the overall meta-analyses. This might be primarily because the patients' characteristics and treatment selection were different among studies. We attempted to conduct the subgroup analyses to explore the sources of heterogeneity. For example, as for pre-operative TACE, the heterogeneity became not significant in the OS subgroup analysis of large HCC, liver cirrhosis, and RCTs, but remained significant in the OS subgroup analysis of small HCC and non-RCTs. In addition, we employed only random-effect models to produce conservative results. Certainly, given such a statistically significant heterogeneity among studies, we had to acknowledge that our conclusions should be cautiously interpreted. Second, a majority of included studies were non-RCTs. To overcome this limitation, we attempted to perform the subgroup meta-analyses according to the study design. Third, the quality of included studies was unsatisfactory. Most of RCTs had the potential risk of bias in the allocation concealment and blinding methods. Notably, TACE was an interventional radiological procedure, rather than a drug. Thus, it might be impractical to blind the treatment assignment. Fourth, the information regarding TACE techniques, anticancer drugs, and embolization drugs was lacking or heterogeneous among studies. Thus, we could not compare the difference among them. Fifth, most of included papers regarding post-operative TACE were from oriental countries. Some of them were published in Chinese language, so the original data were hardly understood by Western readers. Certainly, their primary items were selectively shown in our paper.

In conclusion, based on the present systematic review and meta-analysis, pre-operative TACE should not be considered as an adjunctive treatment option of HCC. But it should be never neglected that pre-operative TACE can lead to complete necrosis of HCC in selected cases, thereby improving the DFS after hepatic resection. Thus, we should further identify the candidates who are potentially eligible for pre-operative TACE. On the other hand, post-operative TACE should be recommended, especially in patients with more advanced HCC treated with hepatic resection. However, due to the relatively poor quality of included studies, well-designed RCTs should be warranted to confirm these findings.

## MATERIALS AND METHODS

This work was registered with PROSPERO (registration number: CRD42015019207).

### Search strategy

The PubMed, EMBASE, and Cochrane Library databases were searched. As previously described [83], search items were as follows: (“hepatectomy” OR “liver resection” OR “hepatic resection” OR “liver surgery” OR “hepatic surgery”) AND (“TACE” OR “transarterial chemoembolization”) AND (“HCC” OR “hepatocellular carcinoma” OR “hepatic carcinoma”). The last search was performed on December 18, 2014. Relevant literatures were also manually searched.

### Study selection

The inclusion criteria should be as follows.

*Participants*: patients should be diagnosed with HCC irrespectively of tumor stage.

*Interventions*: in the experimental group, patients should undergo hepatic resection in combination with pre-operative or post-operative TACE; and in the control group, patients should undergo hepatic resection alone. If hepatic resection was performed in combination with systemic chemotherapy or transarterial infusion of chemotherapy rather than TACE, they would not be considered as experimental groups. Additionally, it should be noted that post-operative TACE should be prophylactic but not therapeutic. In other words, if TACE was employed for the treatment of recurrent HCC or residual tumor after hepatic resection, it would not be considered as experimental groups. The interval between TACE and hepatic resection was not arbitrarily restricted.

*Comparisons*: the outcomes should be compared between patients undergoing hepatic resection in combination with and without TACE. There were two different conditions according to the timing of TACE and hepatic resection. They should include hepatic resection in combination with pre-operative TACE versus hepatic resection alone and hepatic resection in combination with post-operative TACE versus hepatic resection alone.

*Outcomes*: the outcomes observed should include OS, recurrence-free survival or DFS, time-to-recurrence, and/or recurrence rate. Notably, both recurrence-free survival and DFS were considered as the same outcome “DFS”.

The exclusion criteria should be as follows.

1. Duplicate papers among databases or redundant publications [84].
2. Narrative or systematic reviews or study protocols.
3. Comments.
4. Experimental studies.
5. Case reports.
6. Hepatic metastases.
7. Mixed malignancies.
8. Non-comparative studies.
9. No comparison between hepatic resection versus TACE.
10. Comparison between hepatic resection versus TACE for HCC.
11. No separate data in two groups.
12. No detailed data regarding the survival rate in two groups.

Type of study design was not restricted. Either randomized or non-randomized studies were eligible in the systematic review. Publication status and language were not restricted. If two or more papers by the same study team had the overlapping data, only one paper with more adequate data and/or a longer enrollment period would be included.

### Data extraction

The following data were extracted: the first author, publication year, publication form, region, enrollment period, study design, study population, follow-up time, inclusion and exclusion criteria, number of HCC cases, timing of TACE, interval between TACE and hepatic resection, OS rate, DFS rate, rate free of recurrence, and their corresponding Kaplan-Meier curve analyses with log-rank test. If the propensity score matching analysis was performed, we just collected the survival data after the propensity score matching analyses. If both survival rates and Kaplan-Meier curves were presented, only the survival rates would be collected. If only Kaplan-Meier curves were presented, we extracted the cumulative 1-, 3-, and 5-year survival rates by using the Distance Tool in the Measurements menu of Foxit PDF Reader software (Foxit Cooperation, California, USA). This software was freely downloaded.

### Study quality

The quality of cohort and case-control studies was evaluated according to the Newcastle-Ottawa scales for the cohort and case-control studies, respectively [85]. Newcastle-Ottawa scale was composed of 3 major sections with 8 questions, such as Selection section with 4 questions, Comparability section with 1 question, and Exposure section with 3 questions. A study can be given a maximum of 1 point for each question within the Selection and Exposure sections, and a maximum of 2 points for the sole question within the Comparability section.

The 8 relevant questions for cohort studies were as follows:

1. Selection section: representativeness of hepatic resection in combination with TACE group.
2. Selection section: selection of hepatic resection alone group.
3. Selection section: ascertainment of hepatic resection in combination with TACE group.
4. Selection section: demonstration that outcome of interest was not present at start of study.
5. Comparability section: comparability of cohorts on the basis of the design or analysis.
6. Outcome section: assessment of outcome.
7. Outcome section: was follow-up long enough for outcomes to occur.
8. Outcome section: adequacy of follow up of cohorts.

The 8 relevant questions for case-control studies were as follows:

1. Selection section: definition of hepatic resection in combination with TACE group.
2. Selection section: representativeness of hepatic resection in combination with TACE group.
3. Selection section: selection of hepatic resection alone group.
4. Selection section: definition of hepatic resection alone group.
5. Comparability section: comparability of cohorts on the basis of the design or analysis.
6. Outcome section: ascertainment of outcome.
7. Outcome section: same method of ascertainment for cases and controls.
8. Outcome section: non-response rate.

The quality of RCTs was evaluated according to the Cochrane Collaboration's tool for assessing the risk of bias. This tool was composed of 6 sections, such as random sequence generation (selection bias), allocation concealment (selection bias), blinding of participants and personnel (performance bias), blinding of outcome assessment (detection bias), incomplete outcome data addressed (attrition bias), and selective reporting (reporting bias). “High risk”, “low risk”, or “unclear risk” was given to every section.

### Data analysis

Meta-analyses were performed by the statistical package Review Manager version 5.3.5 (Copenhagen, The Nordic Cochrane Center, The Cochrane Collaboration, 2014). Only random-effects models were employed. Because the OS, DFS, and rate free of recurrence were the time-dependent data, the HRs with 95%CIs were pooled by using the calculation sheets developed by Tierney et al. [86]. Heterogeneity between studies was assessed by using the I^2^ statistic and the Chi-square test. I^2^ >50% or *P* < 0.10 was considered to represent a significant heterogeneity. Funnel plots were performed to evaluate the publication bias. If all studies laid within 95%CI, there was no proof of publication bias. Otherwise, there was a proof of publication bias. As for the pre-operative TACE, the subgroup meta-analyses were performed according to the tumor necrosis after TACE (complete tumor necrosis versus incomplete or no tumor necrosis), tumor size (large HCC versus small HCC), liver cirrhosis (cirrhotic versus non-cirrhotic), and type of study design (RCT versus non-RCT). Generally, large HCC was arbitrarily defined as the largest diameter of HCC was >5 cm; by contrast, small HCC was defined as the largest diameter of HCC was ≤5 cm. Additionally, the definitions of large and small HCC were extracted and followed according to every included paper. As for the post-operative TACE, the subgroup meta-analyses were performed according to the vascular invasion (vascular invasion versus no vascular invasion or extrahepatic spread), tumor size (large HCC versus small HCC), liver cirrhosis (cirrhotic versus non-cirrhotic), and type of study design (RCT versus non-RCT). Subgroup difference was assessed by using the I^2^ statistic and the Chi-square test. I^2^> 50% or *P* < 0.10 was considered as having a significant subgroup difference.

## SUPPLEMENTARY FIGURES AND TABLES


