# Supplementary material for: Hepatic resection alone versus in combination with pre- and post-operative transarterial chemoembolization for the treatment of hepatocellular carcinoma: A systematic review and meta-analysis
Source: Oncotarget. 2015 Oct 5;6(34):36838–59. doi: 10.18632/oncotarget.5426 (PMC4742214; doi:10.18632/oncotarget.5426)
Supplement: Supplementary file 1 [file oncotarget-06-36838-s001.pdf]

SUPPLEMENTARY FIGURES AND TABLES

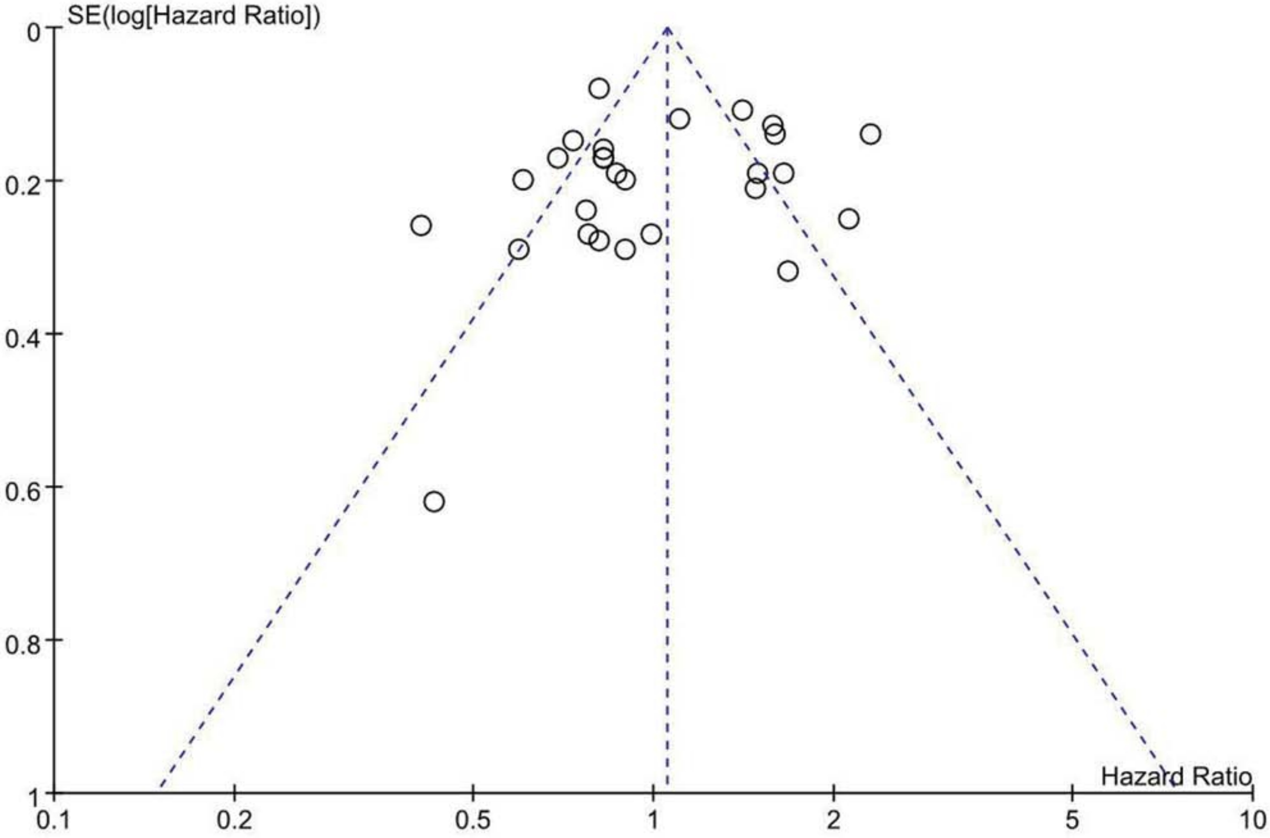

Supplementary Figure S1: Funnel plot regarding the comparison of overall survival between hepatic resection with and without pre-operative TACE groups.

Study or Subgroup	log[Hazard Ratio]	SE	Pre-operative TACE Total	NoTACE Total	Weight	Hazard Ratio IV, Random, 95% CI
**1.2.1 Complete tumor necrosis after TACE**						
Harada T 1996 - subgroup 1	-0.3	0.38	29	33	9.8%	0.74 [0.35, 1.56]
Kim IS 2008	0.24	0.21	52	237	12.0%	1.27 [0.84, 1.92]
Kishi Y 2012	0.88	0.51	8	158	8.1%	2.41 [0.89, 6.55]
Nishikawa H 2013	-0.41	0.23	85	125	11.7%	0.66 [0.42, 1.04]
**Subtotal (95% CI)**			**174**	**553**	**41.6%**	**1.02 [0.63, 1.66]**
Heterogeneity: Tau² = 0.15; Chi² = 8.07, df = 3 (P = 0.04); I² = 63%						
Test for overall effect: Z = 0.08 (P = 0.93)						
**1.2.2 Incomplete or no tumor necrosis after TACE**						
Harada T 1996 - subgroup 2	0.02	0.28	42	33	11.1%	1.02 [0.59, 1.77]
Harada T 1996 - subgroup 3	-0.2	0.33	27	33	10.5%	0.82 [0.43, 1.56]
Kim IS 2008	0.98	0.19	45	237	12.2%	2.66 [1.84, 3.87]
Kishi Y 2012	1.21	0.18	61	158	12.3%	3.35 [2.36, 4.77]
Nishikawa H 2013	1.21	0.18	61	0	12.3%	3.35 [2.36, 4.77]
**Subtotal (95% CI)**			**236**	**461**	**58.4%**	**2.01 [1.22, 3.31]**
Heterogeneity: Tau² = 0.27; Chi² = 27.10, df = 4 (P < 0.0001); I² = 85%						
Test for overall effect: Z = 2.73 (P = 0.006)						
**Total (95% CI)**			**410**	**1014**	**100.0%**	**1.52 [0.97, 2.40]**
Heterogeneity: Tau² = 0.41; Chi² = 65.26, df = 8 (P < 0.00001); I² = 88%						
Test for overall effect: Z = 1.81 (P = 0.07)						
Test for subgroup differences: Chi² = 3.60, df = 1 (P = 0.06), I² = 72.2%						

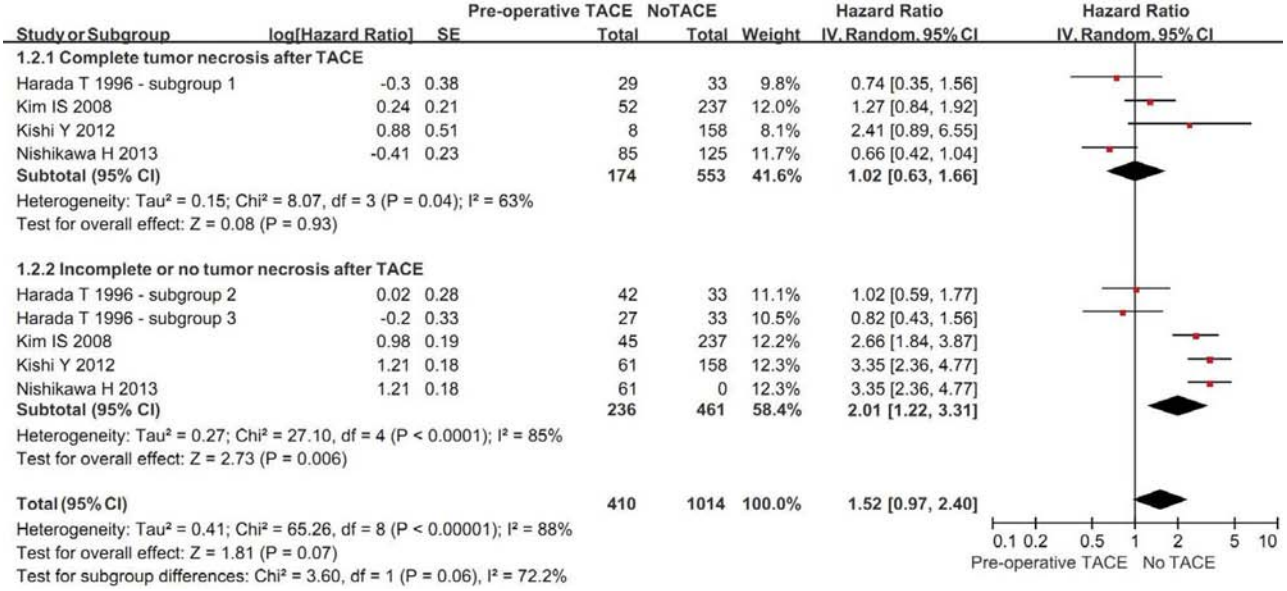

Supplementary Figure S2: Subgroup meta-analysis comparing the overall survival between hepatic resection with and without pre-operative TACE groups according to the tumor necrosis.

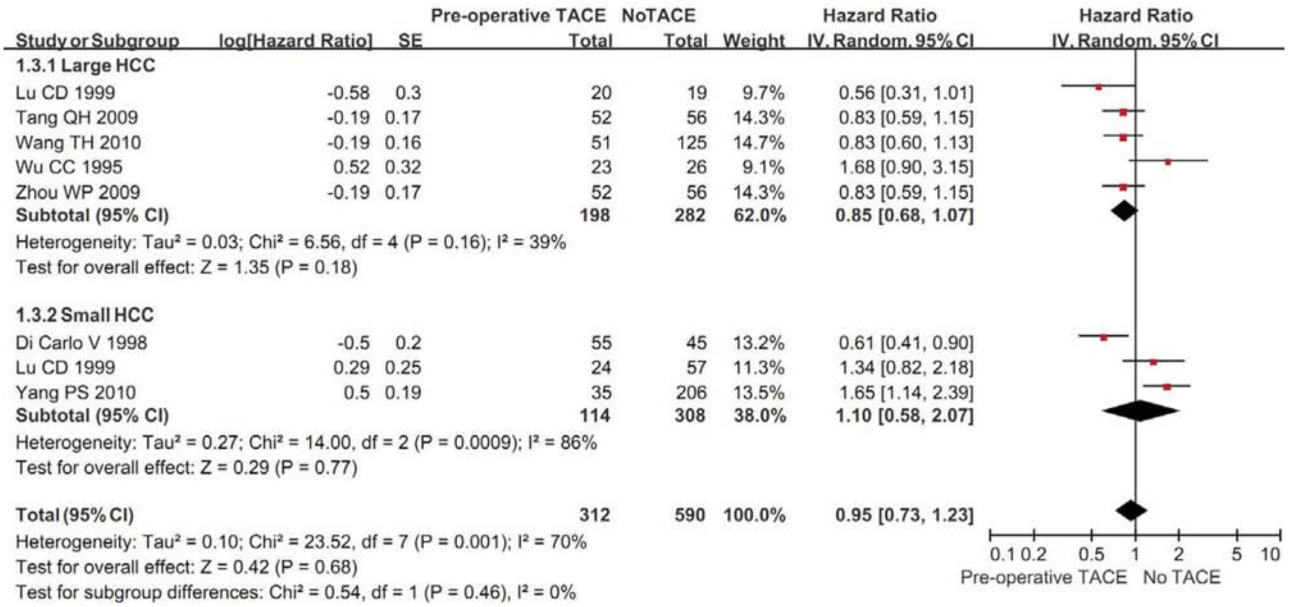

Supplementary Figure S3: Subgroup meta-analysis comparing the overall survival between hepatic resection with and without pre-operative TACE groups according to the tumor size.

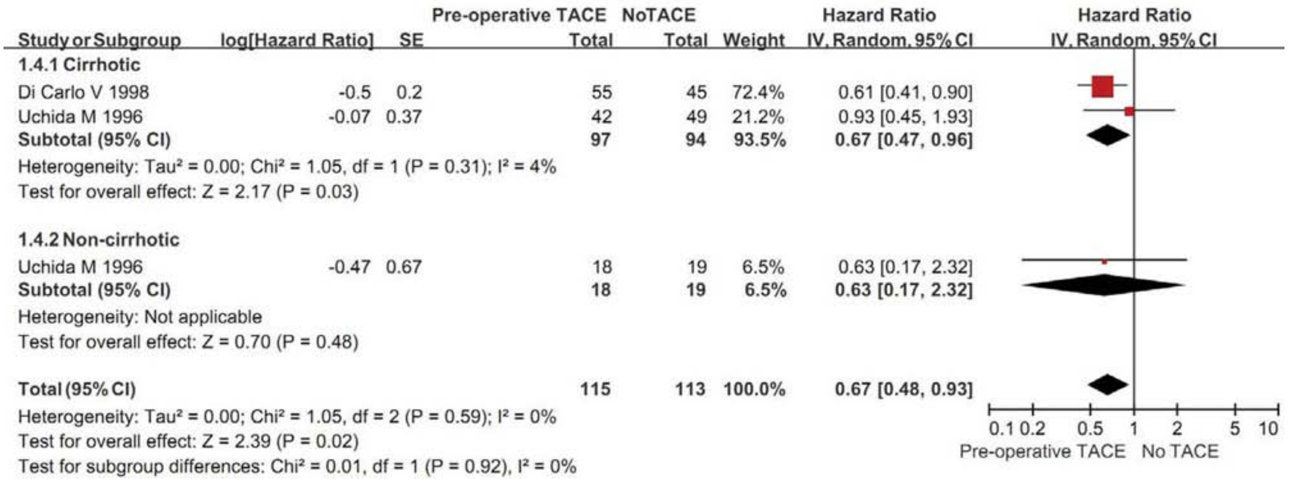

Supplementary Figure S4: Subgroup meta-analysis comparing the overall survival between hepatic resection with and without pre-operative TACE groups according to the presence of liver cirrhosis.

Study or Subgroup	log[Hazard Ratio]	SE	Pre-operative TACE Total	NoTACE Total	Weight	Hazard Ratio IV, Random, 95% CI
**1.5.1 RCT**						
Kaibori M 2012	-0.21	0.28	81	43	3.1%	0.81 [0.47, 1.40]
Tang QH 2009	-0.19	0.17	52	56	4.1%	0.83 [0.59, 1.15]
Wu CC 1995	0.52	0.32	23	26	2.8%	1.68 [0.90, 3.15]
Yamasaki S 1996	-0.11	0.29	50	47	3.0%	0.90 [0.51, 1.58]
Zhou WP 2009	-0.19	0.17	52	56	4.1%	0.83 [0.59, 1.15]
**Subtotal (95% CI)**			**258**	**228**	**17.0%**	**0.90 [0.73, 1.10]**
Heterogeneity: Tau² = 0.01; Chi² = 4.44, df = 4 (P = 0.35); I² = 10%						
Test for overall effect: Z = 1.04 (P = 0.30)						
**1.5.2Non-RCT**						
Chen XP 2007	-0.31	0.15	89	157	4.2%	0.73 [0.55, 0.98]
Di Carlo V 1998	-0.5	0.2	55	45	3.8%	0.61 [0.41, 0.90]
Harada T 1996	-0.52	0.29	98	33	3.0%	0.59 [0.34, 1.05]
Jianyong L 2014	0.1	0.12	183	405	4.5%	1.11 [0.87, 1.40]
Kaibori M 2006 - subgroup 1	-0.25	0.27	49	57	3.2%	0.78 [0.46, 1.32]
Kaibori M 2006 - subgroup 2	-0.89	0.26	66	53	3.3%	0.41 [0.25, 0.68]
Kang JY 2008	0.39	0.21	32	64	3.7%	1.48 [0.98, 2.23]
Kim IS 2008	0.47	0.14	97	237	4.3%	1.60 [1.22, 2.11]
Kishi Y 2012	0.83	0.14	69	158	4.3%	2.29 [1.74, 3.02]
Liu YJ 2010	0.46	0.13	71	156	4.4%	1.58 [1.23, 2.04]
Lu CD 1999	-0.14	0.19	44	76	3.9%	0.87 [0.60, 1.26]
Nagasue N 1989	0.4	0.19	31	107	3.9%	1.49 [1.03, 2.16]
Nishikawa H 2013	-0.11	0.2	110	125	3.8%	0.90 [0.61, 1.33]
Ochiai T 2003	-0.26	0.24	100	48	3.4%	0.77 [0.48, 1.23]
Paye F 1998	-0.84	0.62	24	24	1.3%	0.43 [0.13, 1.46]
Sasaki A 2006	0.34	0.11	109	126	4.5%	1.40 [1.13, 1.74]
Shi HY 2014	-0.21	0.08	648	648	4.7%	0.81 [0.69, 0.95]
Uchida M 1996	-0.01	0.27	60	68	3.2%	0.99 [0.58, 1.68]
Wang TH 2010	-0.19	0.16	51	125	4.2%	0.83 [0.60, 1.13]
Xiao EH 2005	-0.37	0.17	81	58	4.1%	0.69 [0.50, 0.96]
Yanaga K 2014	0.75	0.25	37	176	3.4%	2.12 [1.30, 3.46]
Yang PS 2010	0.5	0.19	35	206	3.9%	1.65 [1.14, 2.39]
**Subtotal (95% CI)**			**2139**	**3152**	**83.0%**	**1.03 [0.86, 1.23]**
Heterogeneity: Tau² = 0.14; Chi² = 132.05, df = 21 (P < 0.00001); I² = 84%						
Test for overall effect: Z = 0.29 (P = 0.77)						
**Total (95% CI)**			**2397**	**3380**	**100.0%**	**1.01 [0.87, 1.19]**
Heterogeneity: Tau² = 0.13; Chi² = 139.71, df = 26 (P < 0.00001); I² = 81%						
Test for overall effect: Z = 0.16 (P = 0.87)						
Test for subgroup differences: Chi² = 0.95, df = 1 (P = 0.33), I² = 0%						

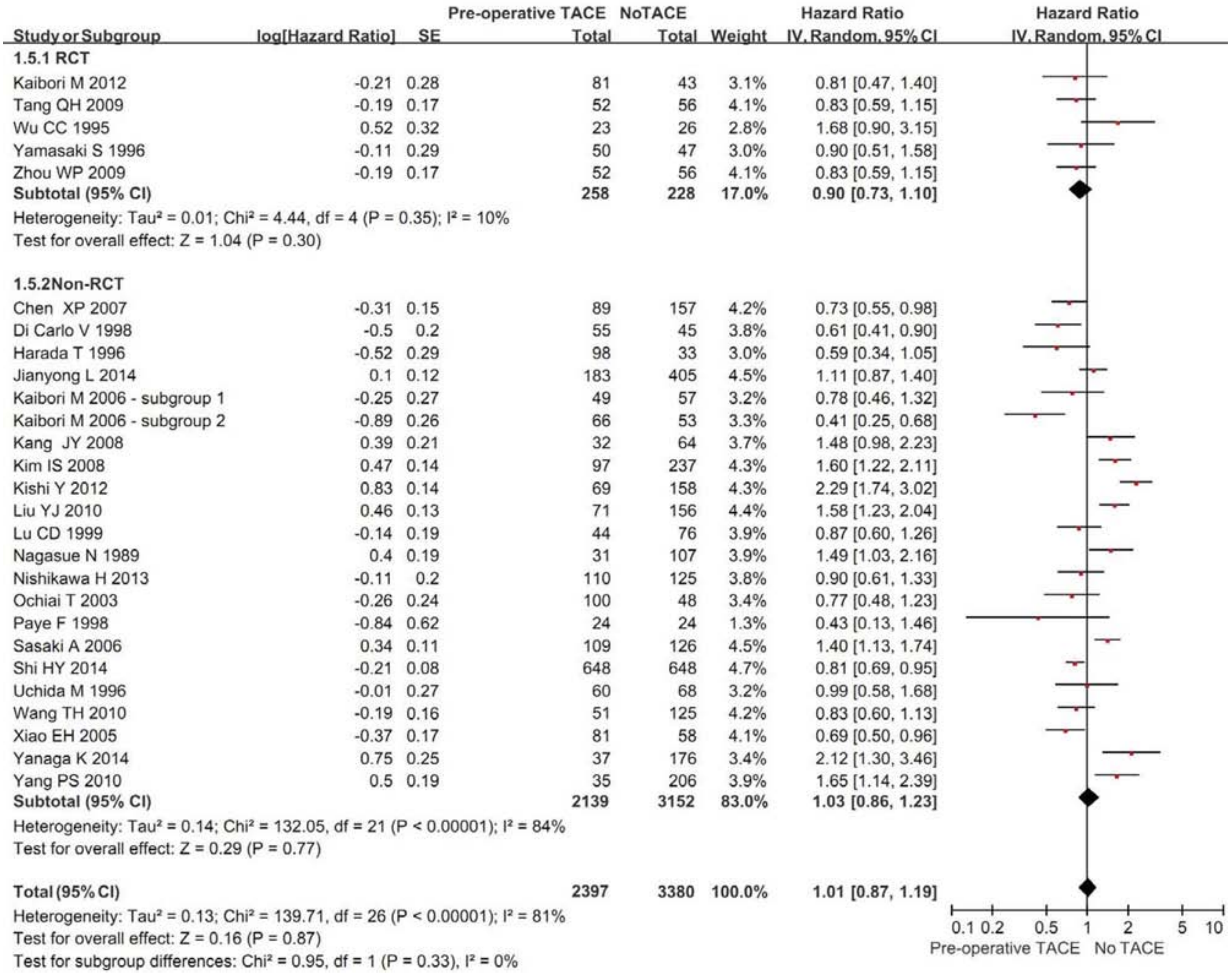

Supplementary Figure S5: Subgroup meta-analysis comparing the overall survival between hepatic resection with and without pre-operative TACE groups according to the study design.

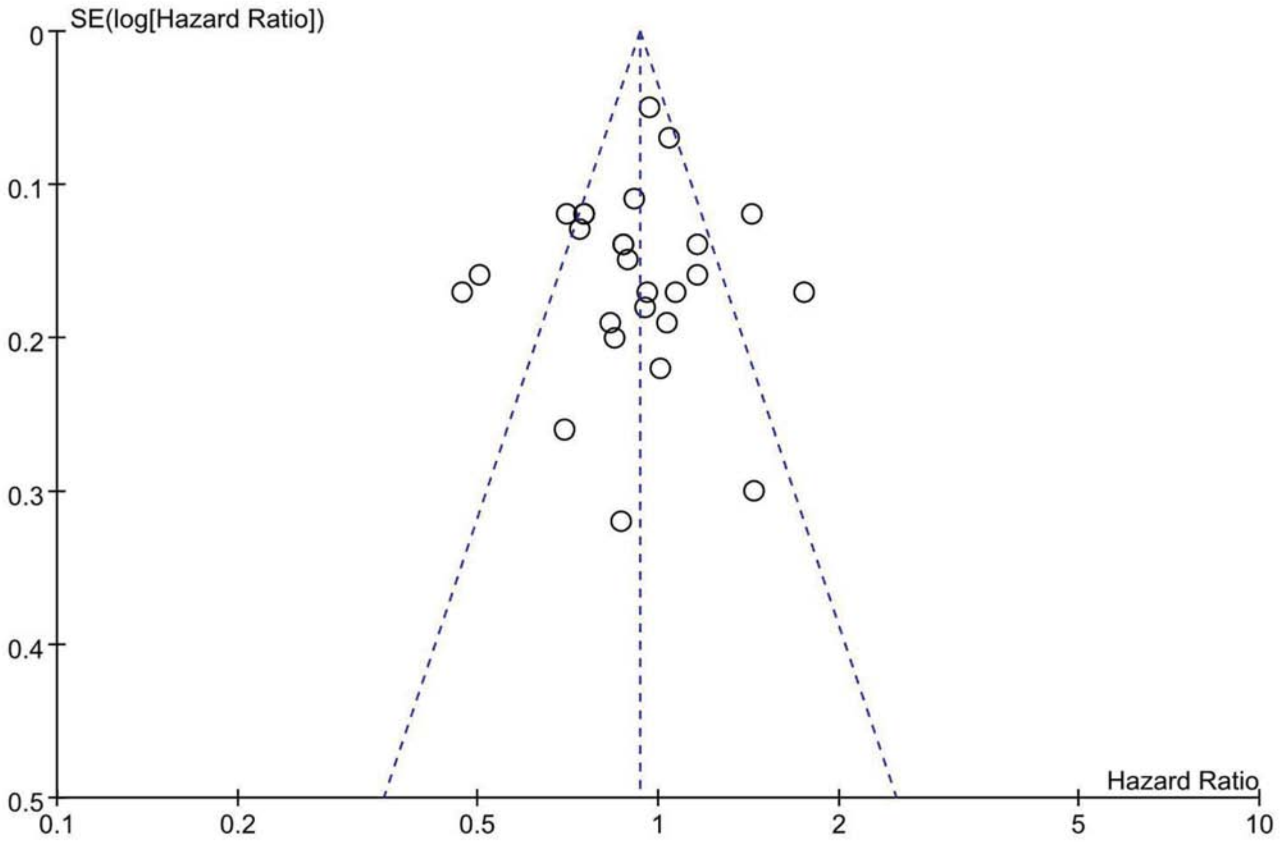

Supplementary Figure S6: Funnel plot regarding the comparison of disease-free survival between hepatic resection with and without pre-operative TACE groups.

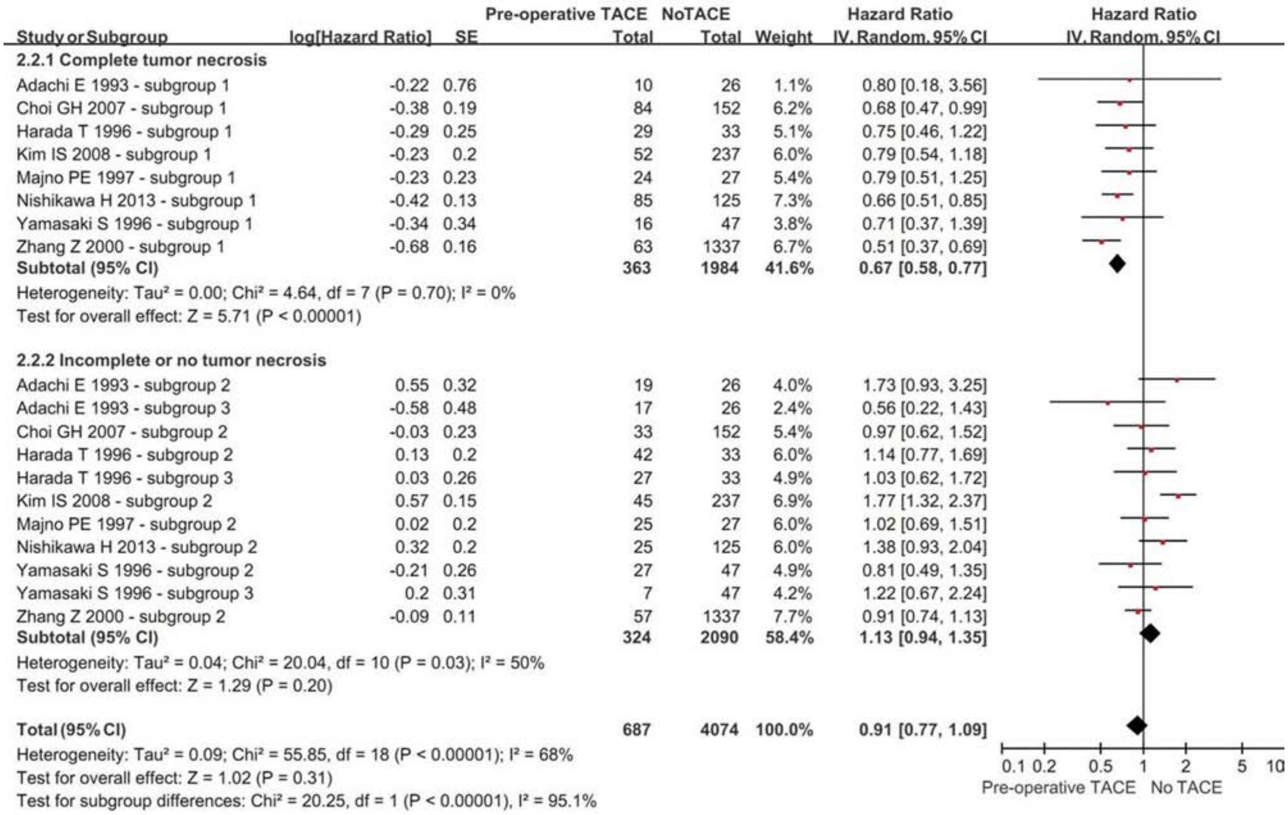

Supplementary Figure S7: Subgroup meta-analysis comparing the disease-free survival between hepatic resection with and without pre-operative TACE groups according to the tumor necrosis.

Study or Subgroup	log[Hazard Ratio]	SE	Pre-operative TACE Total	NoTACE Total	Weight	Hazard Ratio IV, Random, 95% CI
**2.3.1 Large HCC**						
Choi GH 2007 - subgroup 1	-0.32	0.24	44	49	8.4%	0.73 [0.45, 1.16]
Lu CD 1999 - subgroup 1	-0.6	0.3	20	19	6.6%	0.55 [0.30, 0.99]
Tang QH 2009	-0.13	0.14	52	56	12.0%	0.88 [0.67, 1.16]
Wu CC 1995	0.37	0.3	23	26	6.6%	1.45 [0.80, 2.61]
Zhou WP 2009	-0.13	0.14	52	56	12.0%	0.88 [0.67, 1.16]
**Subtotal (95% CI)**			**191**	**206**	**45.6%**	**0.86 [0.69, 1.06]**
Heterogeneity: Tau² = 0.02; Chi² = 5.80, df = 4 (P = 0.21); I² = 31%						
Test for overall effect: Z = 1.44 (P = 0.15)						
**2.3.2 Small HCC**						
Adachi E 1993	-0.14	0.32	46	26	6.2%	0.87 [0.46, 1.63]
Choi GH 2007 - subgroup 2	0.26	0.32	30	59	6.2%	1.30 [0.69, 2.43]
Choi GH 2007 - subgroup 3	-0.12	0.24	43	44	8.4%	0.89 [0.55, 1.42]
Di Carlo V 1998	-0.3	0.13	55	45	12.4%	0.74 [0.57, 0.96]
Lu CD 1999 - subgroup 2	0.5	0.23	24	57	8.7%	1.65 [1.05, 2.59]
Yang PS 2010	0.36	0.12	35	206	12.7%	1.43 [1.13, 1.81]
**Subtotal (95% CI)**			**233**	**437**	**54.4%**	**1.10 [0.80, 1.50]**
Heterogeneity: Tau² = 0.10; Chi² = 18.80, df = 5 (P = 0.002); I² = 73%						
Test for overall effect: Z = 0.58 (P = 0.56)						
**Total (95% CI)**			**424**	**643**	**100.0%**	**0.98 [0.80, 1.19]**
Heterogeneity: Tau² = 0.07; Chi² = 29.08, df = 10 (P = 0.001); I² = 66%						
Test for overall effect: Z = 0.23 (P = 0.82)						
Test for subgroup differences: Chi² = 1.66, df = 1 (P = 0.20), I² = 39.8%						

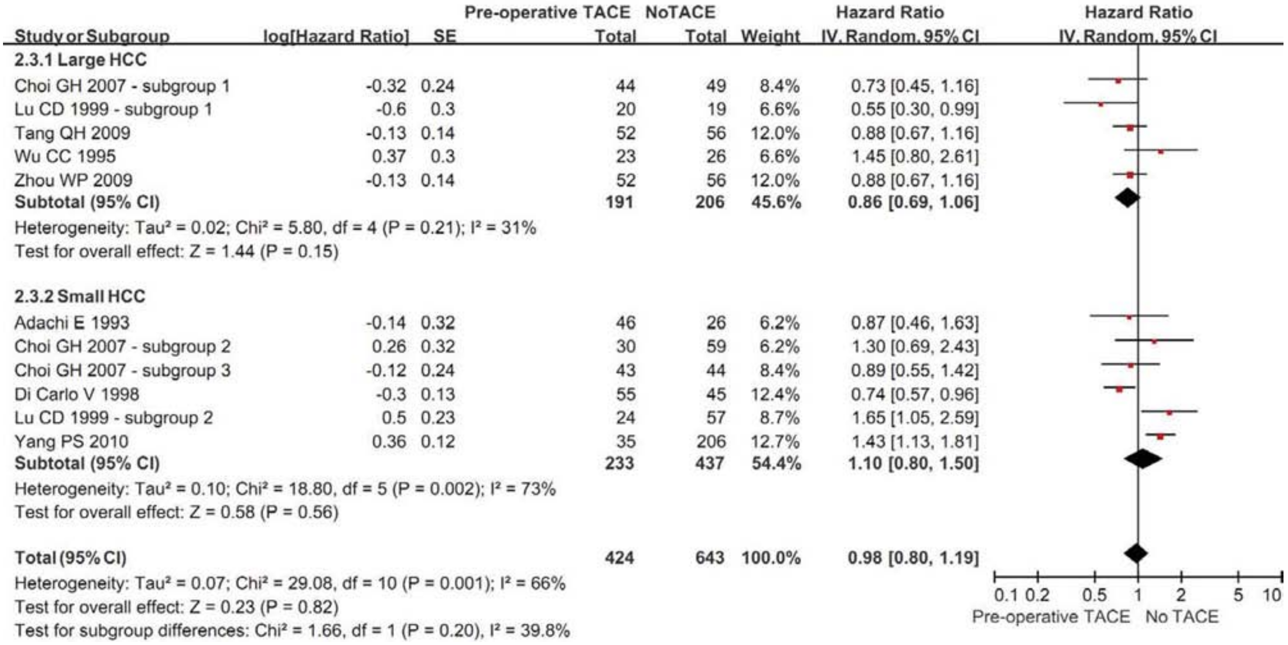

Supplementary Figure S8: Subgroup meta-analysis comparing the disease-free survival between hepatic resection with and without pre-operative TACE groups according to the tumor size.

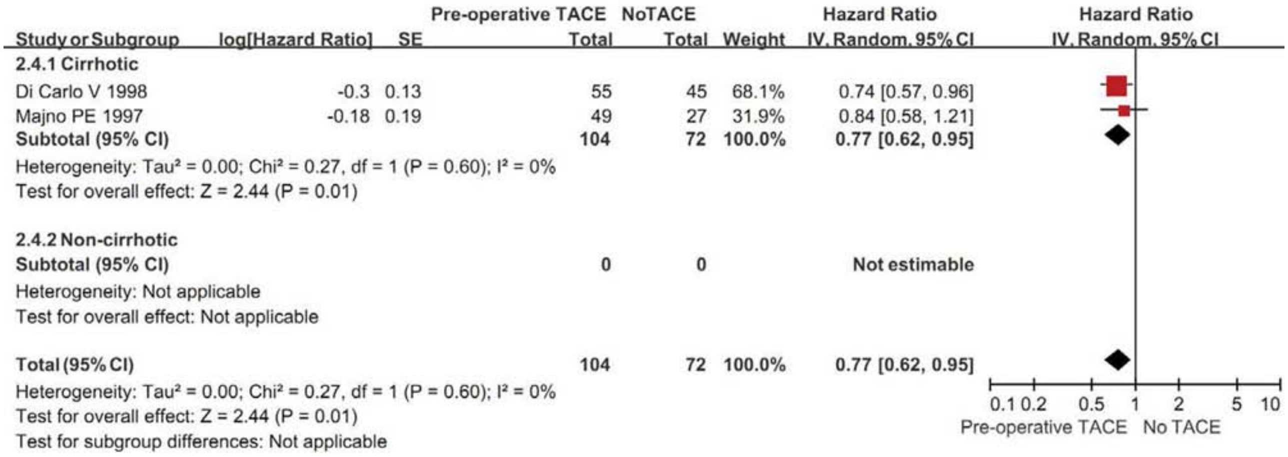

Supplementary Figure S9: Subgroup meta-analysis comparing the disease-free survival between hepatic resection with and without pre-operative TACE groups according to the presence of liver cirrhosis.

Study or Subgroup	log[Hazard Ratio]	SE	Pre-operative TACE Total	NoTACE Total	Weight	Hazard Ratio IV, Random, 95% CI
**2.5.1 RCT**						
Kaibori M 2012	-0.04	0.17	81	43	3.6%	0.96 [0.69, 1.34]
Tang QH 2009	-0.13	0.14	52	56	4.2%	0.88 [0.67, 1.16]
Wu CC 1995	0.37	0.3	23	26	2.0%	1.45 [0.80, 2.61]
Yamasaki S 1996	-0.17	0.2	50	47	3.1%	0.84 [0.57, 1.25]
Zhou WP 2009	-0.13	0.14	52	56	4.2%	0.88 [0.67, 1.16]
**Subtotal (95% CI)**			**258**	**228**	**17.2%**	**0.92 [0.79, 1.07]**
Heterogeneity: Tau² = 0.00; Chi² = 2.76, df = 4 (P = 0.60); I² = 0%						
Test for overall effect: Z = 1.13 (P = 0.26)						
**2.5.2Non-RCT**						
Adachi E 1993	-0.14	0.32	46	26	1.8%	0.87 [0.46, 1.63]
Chen XP 2007	-0.28	0.12	89	157	4.6%	0.76 [0.60, 0.96]
Choi GH 2007	-0.12	0.15	117	152	4.0%	0.89 [0.66, 1.19]
Di Carlo V 1998	-0.3	0.13	55	45	4.4%	0.74 [0.57, 0.96]
Harada T 1996	-0.05	0.18	98	33	3.5%	0.95 [0.67, 1.35]
Jianyong L 2014	0.15	0.16	183	405	3.8%	1.16 [0.85, 1.59]
Kaibori M 2006 - subgroup 1	0.03	0.19	49	57	3.3%	1.03 [0.71, 1.50]
Kaibori M 2006 - subgroup 2	-0.75	0.17	66	53	3.6%	0.47 [0.34, 0.66]
Kang JY 2008	0.56	0.17	32	64	3.6%	1.75 [1.25, 2.44]
Kim IS 2008	0.15	0.14	97	237	4.2%	1.16 [0.88, 1.53]
Lu CD 1999	0.07	0.17	44	76	3.6%	1.07 [0.77, 1.50]
Majno PE 1997	-0.18	0.19	49	27	3.3%	0.84 [0.58, 1.21]
Nishikawa H 2013	-0.35	0.12	110	125	4.6%	0.70 [0.56, 0.89]
Ochiai T 2003	0.01	0.22	100	48	2.9%	1.01 [0.66, 1.55]
Paye F 1998	-0.36	0.26	24	24	2.4%	0.70 [0.42, 1.16]
Sasaki A 2006	0.04	0.07	109	126	5.5%	1.04 [0.91, 1.19]
Shi HY 2014	-0.03	0.05	648	648	5.8%	0.97 [0.88, 1.07]
Sugo H 2003	-0.28	0.12	146	81	4.6%	0.76 [0.60, 0.96]
Yang PS 2010	0.36	0.12	35	206	4.6%	1.43 [1.13, 1.81]
Zhang Z 2000 - subgroup 1	-0.68	0.16	63	1337	3.8%	0.51 [0.37, 0.69]
Zhang Z 2000 - subgroup 2	-0.09	0.11	57	1337	4.8%	0.91 [0.74, 1.13]
**Subtotal (95% CI)**			**2217**	**5264**	**82.8%**	**0.90 [0.81, 1.02]**
Heterogeneity: Tau² = 0.05; Chi² = 82.15, df = 20 (P < 0.00001); I² = 76%						
Test for overall effect: Z = 1.70 (P = 0.09)						
**Total (95% CI)**			**2475**	**5492**	**100.0%**	**0.91 [0.82, 1.01]**
Heterogeneity: Tau² = 0.04; Chi² = 84.94, df = 25 (P < 0.00001); I² = 71%						
Test for overall effect: Z = 1.84 (P = 0.07)						
Test for subgroup differences: Chi² = 0.02, df = 1 (P = 0.88), I² = 0%						

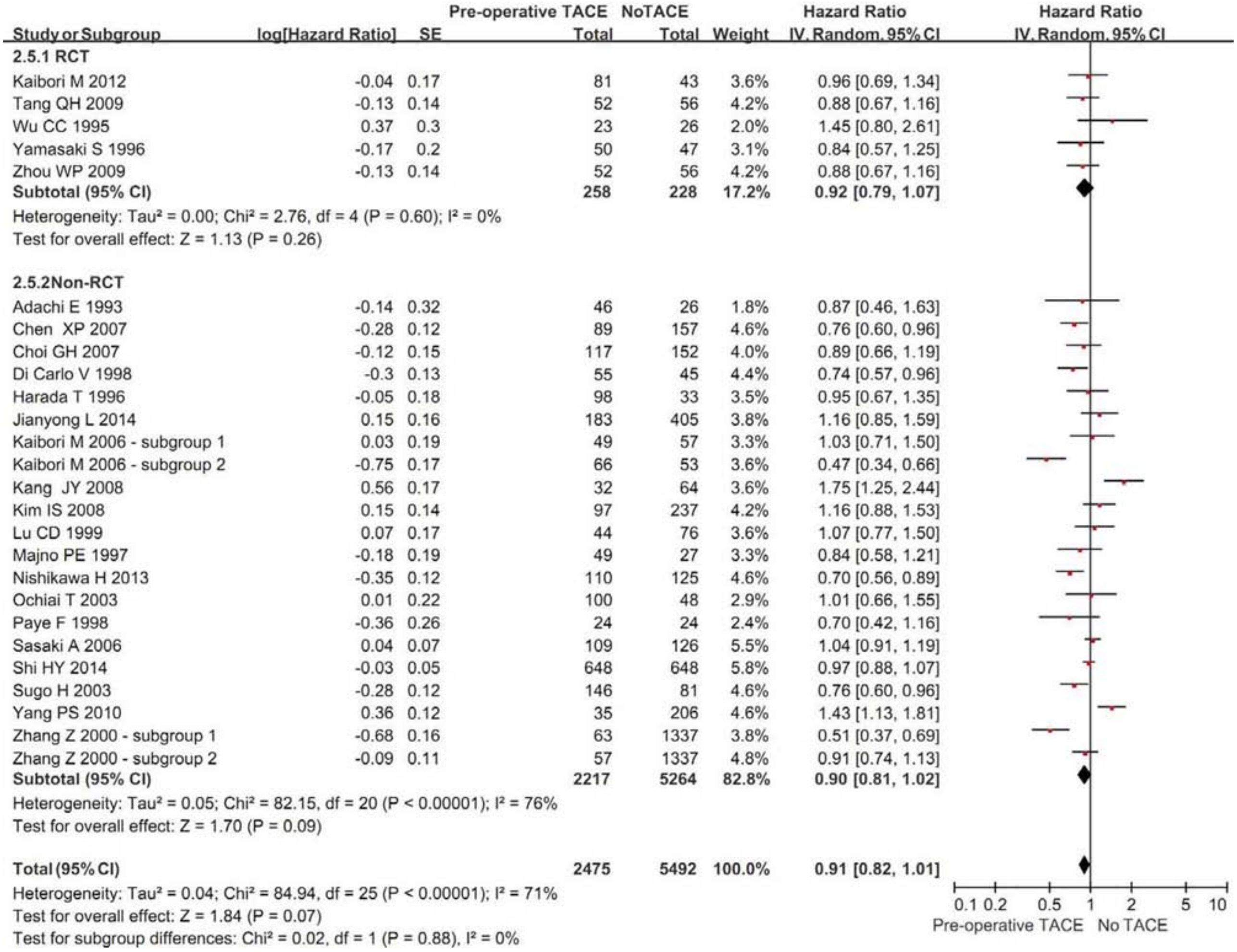

Supplementary Figure S10: Subgroup meta-analysis comparing the disease-free survival between hepatic resection with and without pre-operative TACE groups according to the study design.

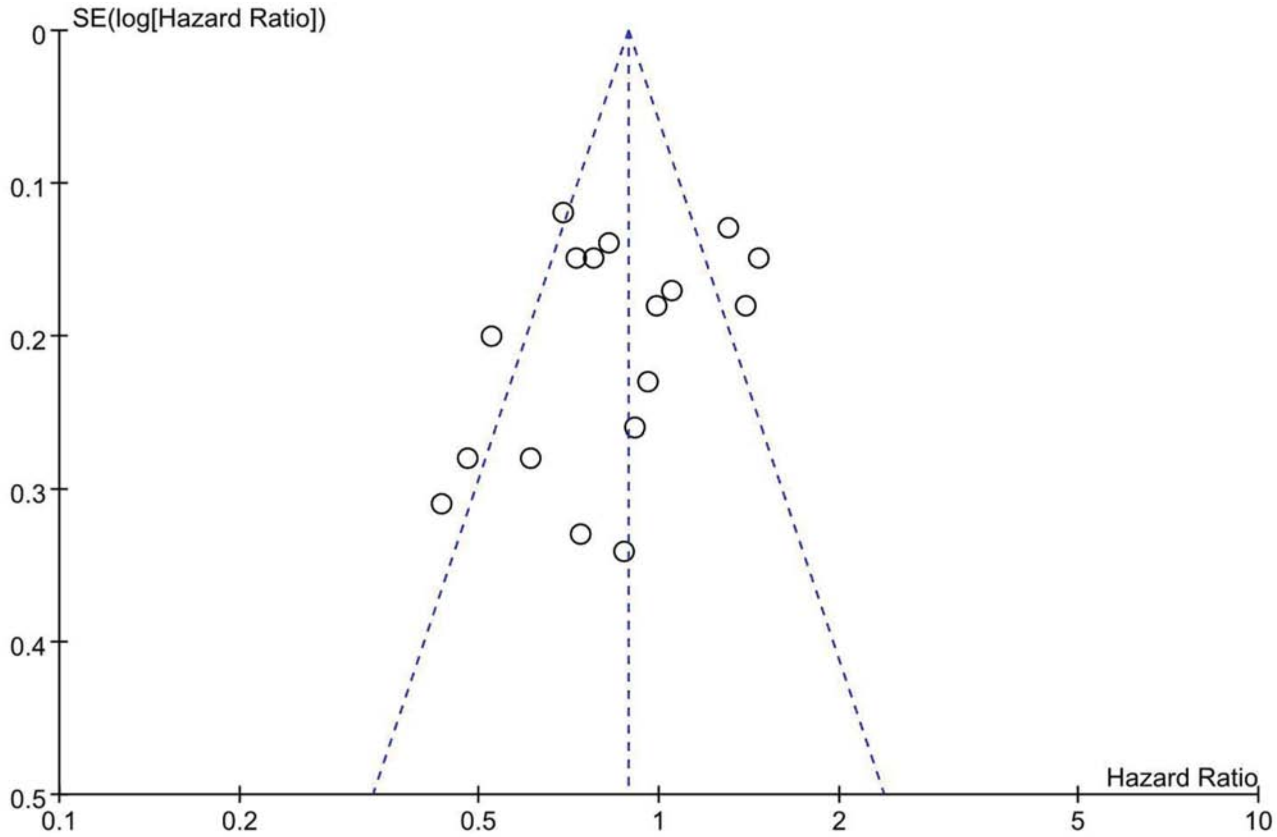

Supplementary Figure S11: Funnel plot regarding the comparison of overall survival between hepatic resection with and without post-operative TACE groups.

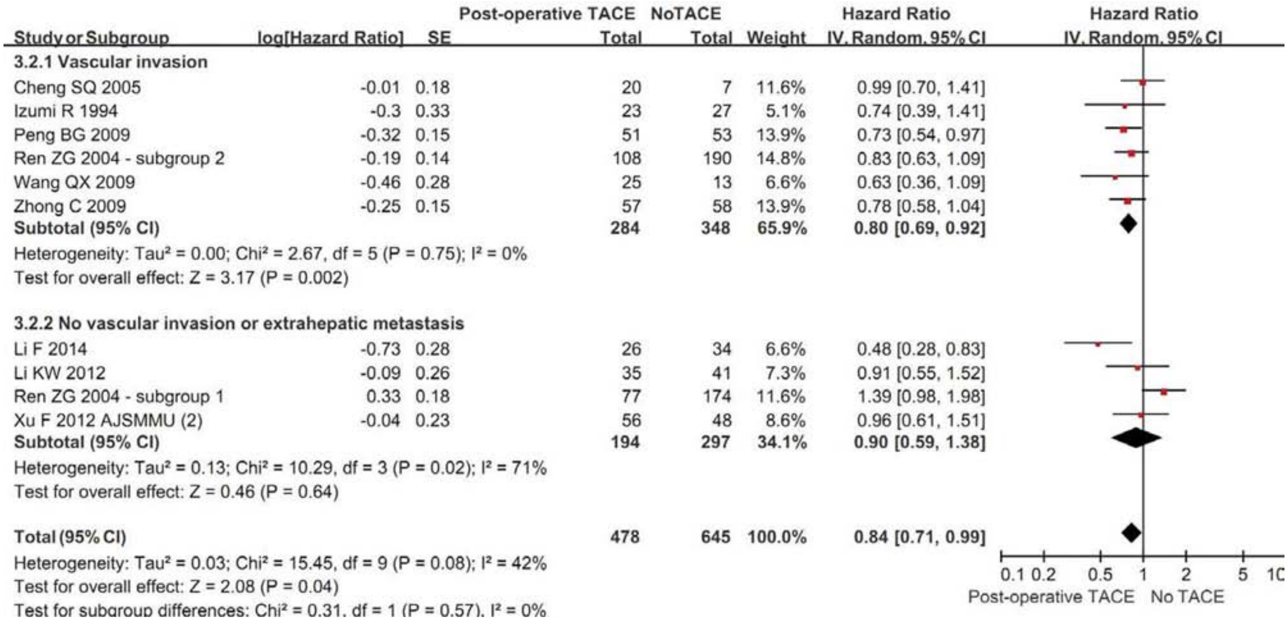

Supplementary Figure S12: Subgroup meta-analysis comparing the overall survival between hepatic resection with and without post-operative TACE groups according to the vascular invasion.

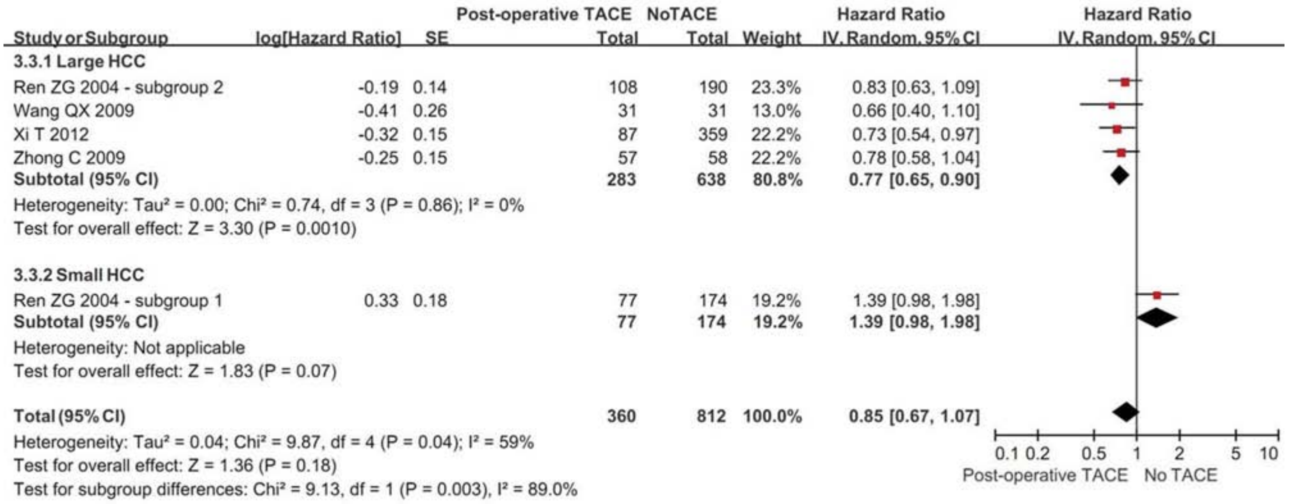

Supplementary Figure S13: Subgroup meta-analysis comparing the overall survival between hepatic resection with and without post-operative TACE groups according to the tumor size.

Study or Subgroup	log[Hazard Ratio]	SE	Post-operative TACE Total	NoTACE Total	Weight	Hazard Ratio IV, Random, 95% CI
**3.4.1 RCT**						
Izumi R 1994	-0.3	0.33	23	27	3.8%	0.74 [0.39, 1.41]
Li JQ 1995	-0.83	0.31	47	47	4.1%	0.44 [0.24, 0.80]
Li Q (DS) 2006	-0.64	0.2	23	23	6.0%	0.53 [0.36, 0.78]
Peng BG 2009	-0.32	0.15	51	53	7.1%	0.73 [0.54, 0.97]
Yu ZP 2009	-0.49	0.28	50	47	4.5%	0.61 [0.35, 1.06]
Zhong C 2009	-0.25	0.15	57	58	7.1%	0.78 [0.58, 1.04]
**Subtotal (95% CI)**			**251**	**255**	**32.6%**	**0.67 [0.57, 0.79]**
Heterogeneity: Tau² = 0.00; Chi² = 4.84, df = 5 (P = 0.44); I² = 0%						
Test for overall effect: Z = 4.88 (P < 0.00001)						
**3.4.2 No RCT**						
Cheng SQ 2005	-0.01	0.18	20	7	6.4%	0.99 [0.70, 1.41]
Gerunda GE 2000	-0.13	0.34	20	17	3.7%	0.88 [0.45, 1.71]
Lee KT 2009	0.38	0.15	114	236	7.1%	1.46 [1.09, 1.96]
Li F 2014	-0.73	0.28	26	34	4.5%	0.48 [0.28, 0.83]
Li KW 2012	-0.09	0.26	35	41	4.9%	0.91 [0.55, 1.52]
Liu YJ 2010	0.27	0.13	86	156	7.5%	1.31 [1.02, 1.69]
Ren ZG 2004 - subgroup 1	0.33	0.18	77	174	6.4%	1.39 [0.98, 1.98]
Ren ZG 2004 - subgroup 2	-0.19	0.14	108	190	7.3%	0.83 [0.63, 1.09]
Wang QX 2009	0.05	0.17	104	156	6.6%	1.05 [0.75, 1.47]
Xi T 2012	-0.37	0.12	145	576	7.7%	0.69 [0.55, 0.87]
Xu F 2012 AJSMMU (2)	-0.04	0.23	56	48	5.4%	0.96 [0.61, 1.51]
**Subtotal (95% CI)**			**791**	**1635**	**67.4%**	**0.98 [0.81, 1.19]**
Heterogeneity: Tau² = 0.07; Chi² = 32.59, df = 10 (P = 0.0003); I² = 69%						
Test for overall effect: Z = 0.22 (P = 0.82)						
**Total (95% CI)**			**1042**	**1890**	**100.0%**	**0.85 [0.72, 1.00]**
Heterogeneity: Tau² = 0.08; Chi² = 53.78, df = 16 (P < 0.00001); I² = 70%						
Test for overall effect: Z = 1.92 (P = 0.06)						
Test for subgroup differences: Chi² = 8.86, df = 1 (P = 0.003), I² = 88.7%						

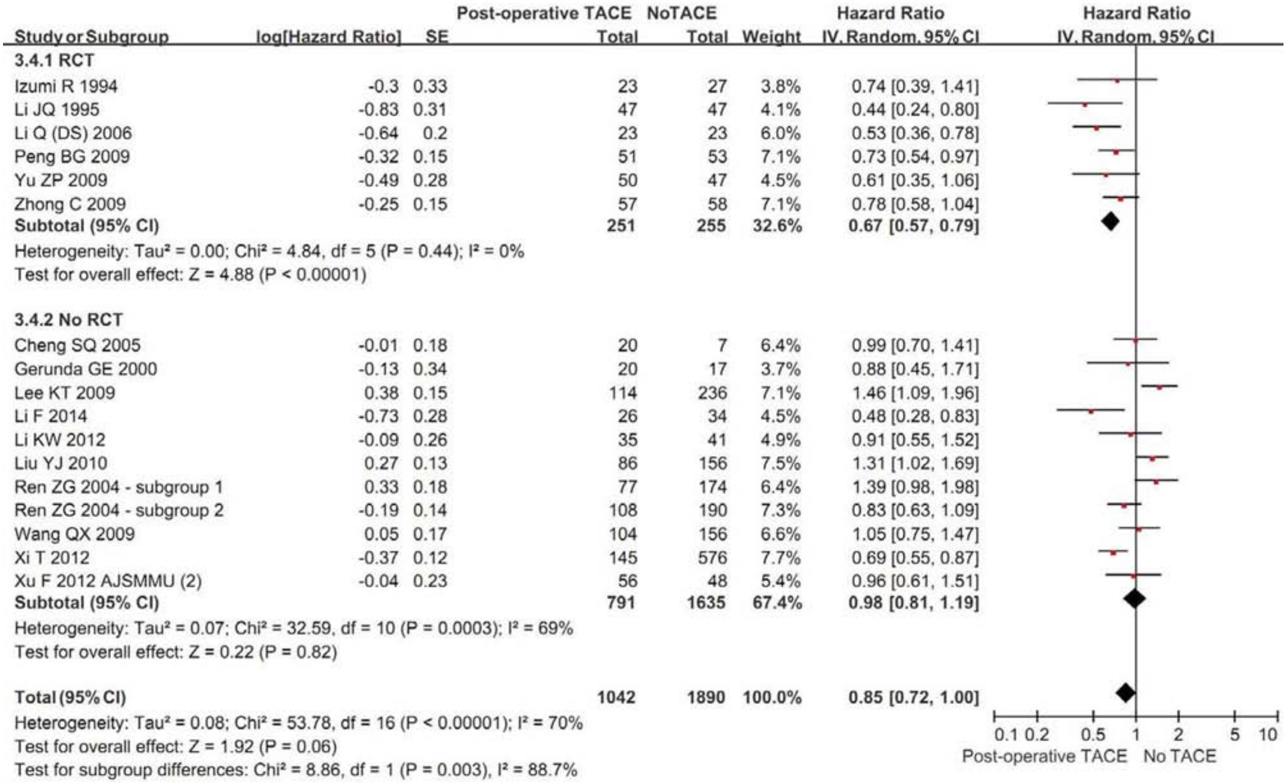

Supplementary Figure S14: Subgroup meta-analysis comparing the overall survival between hepatic resection with and without post-operative TACE groups according to the study design.

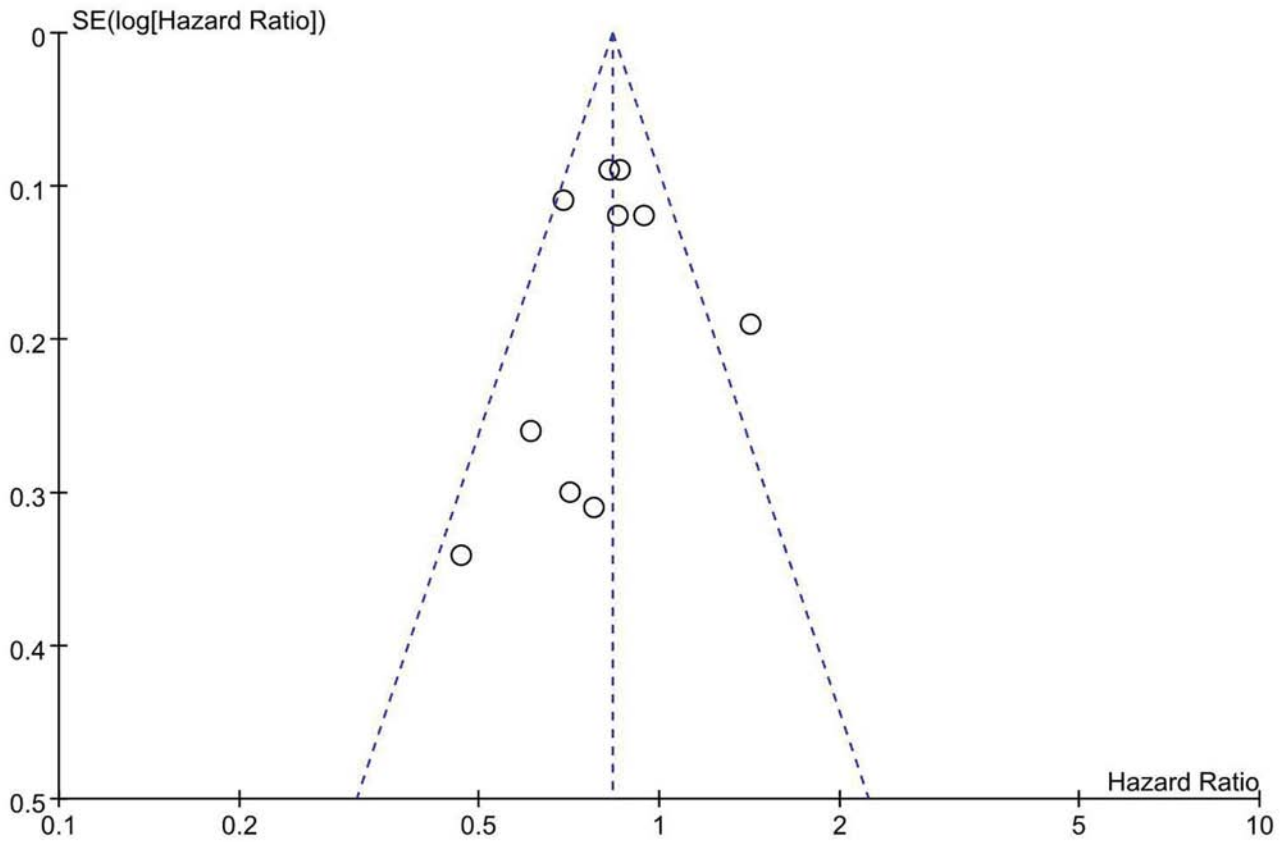

Supplementary Figure S15: Funnel plot regarding the comparison of disease-free survival between hepatic resection with and without post-operative TACE groups.

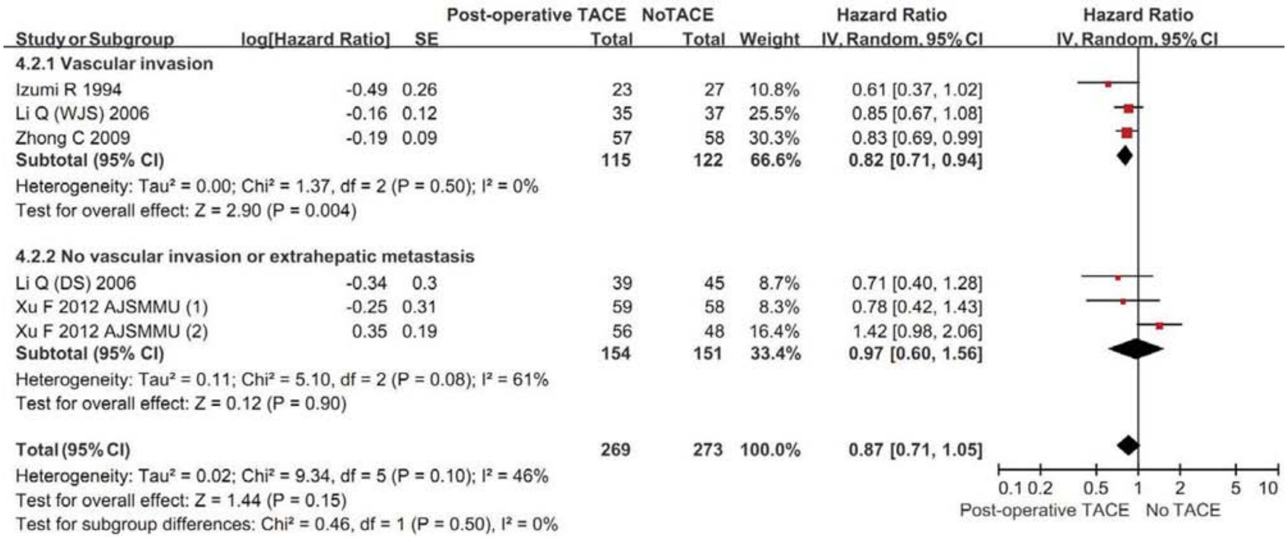

Supplementary Figure S16: Subgroup meta-analysis comparing the disease-free survival between hepatic resection with and without post-operative TACE groups according to the vascular invasion.

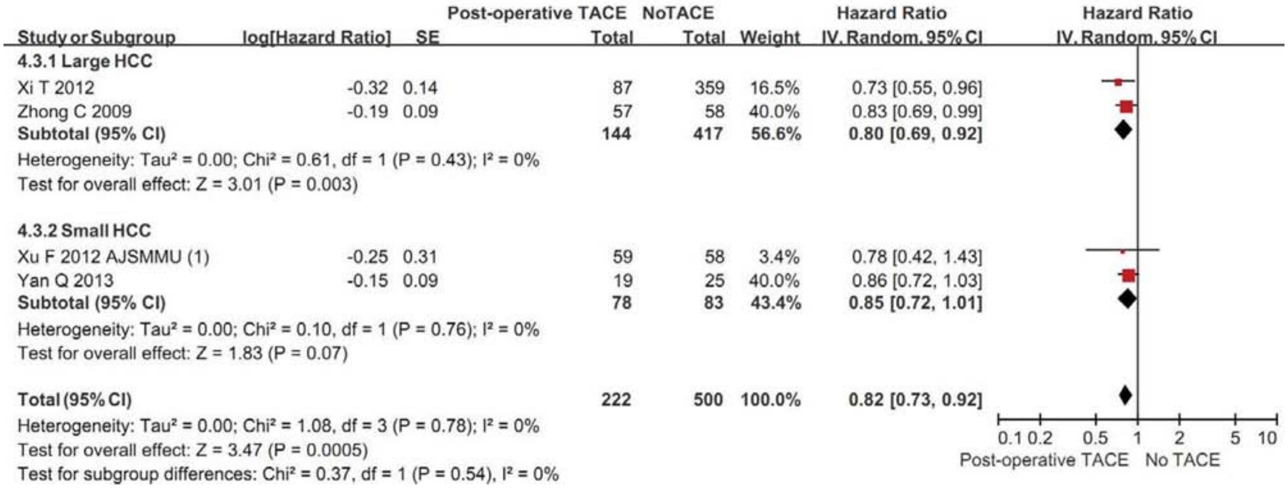

Supplementary Figure S17: Subgroup meta-analysis comparing the disease-free survival between hepatic resection with and without post-operative TACE groups according to the tumor size.

Study or Subgroup	log[Hazard Ratio]	SE	Post-operative TACE Total	NoTACE Total	Weight	Hazard Ratio IV, Random, 95% CI
**4.4.1 RCT**						
Izumi R 1994	-0.49	0.26	23	27	5.0%	0.61 [0.37, 1.02]
Li Q (DS) 2006	-0.34	0.3	39	45	4.0%	0.71 [0.40, 1.28]
Li Q (WJS) 2006	-0.16	0.12	35	37	13.6%	0.85 [0.67, 1.08]
Xu F 2012 AJSMMU (1)	-0.25	0.31	59	58	3.8%	0.78 [0.42, 1.43]
Zhong C 2009	-0.19	0.09	57	58	17.1%	0.83 [0.69, 0.99]
**Subtotal (95% CI)**			**213**	**225**	**43.4%**	**0.81 [0.71, 0.92]**
Heterogeneity: Tau² = 0.00; Chi² = 1.59, df = 4 (P = 0.81); I² = 0%						
Test for overall effect: Z = 3.18 (P = 0.001)						
**4.4.2Non-RCT**						
Gerunda GE 2000	-0.76	0.34	20	17	3.2%	0.47 [0.24, 0.91]
Lee KT 2009	-0.06	0.12	114	236	13.6%	0.94 [0.74, 1.19]
Xi T 2012	-0.37	0.11	145	576	14.7%	0.69 [0.56, 0.86]
Xu F 2012 AJSMMU (1)	0.35	0.19	56	48	8.0%	1.42 [0.98, 2.06]
Yan Q 2013	-0.15	0.09	19	25	17.1%	0.86 [0.72, 1.03]
**Subtotal (95% CI)**			**354**	**902**	**56.6%**	**0.86 [0.68, 1.09]**
Heterogeneity: Tau² = 0.05; Chi² = 14.67, df = 4 (P = 0.005); I² = 73%						
Test for overall effect: Z = 1.24 (P = 0.21)						
**Total (95% CI)**			**567**	**1127**	**100.0%**	**0.83 [0.73, 0.94]**
Heterogeneity: Tau² = 0.02; Chi² = 16.58, df = 9 (P = 0.06); I² = 46%						
Test for overall effect: Z = 2.86 (P = 0.004)						
Test for subgroup differences: Chi² = 0.19, df = 1 (P = 0.66), I² = 0%						

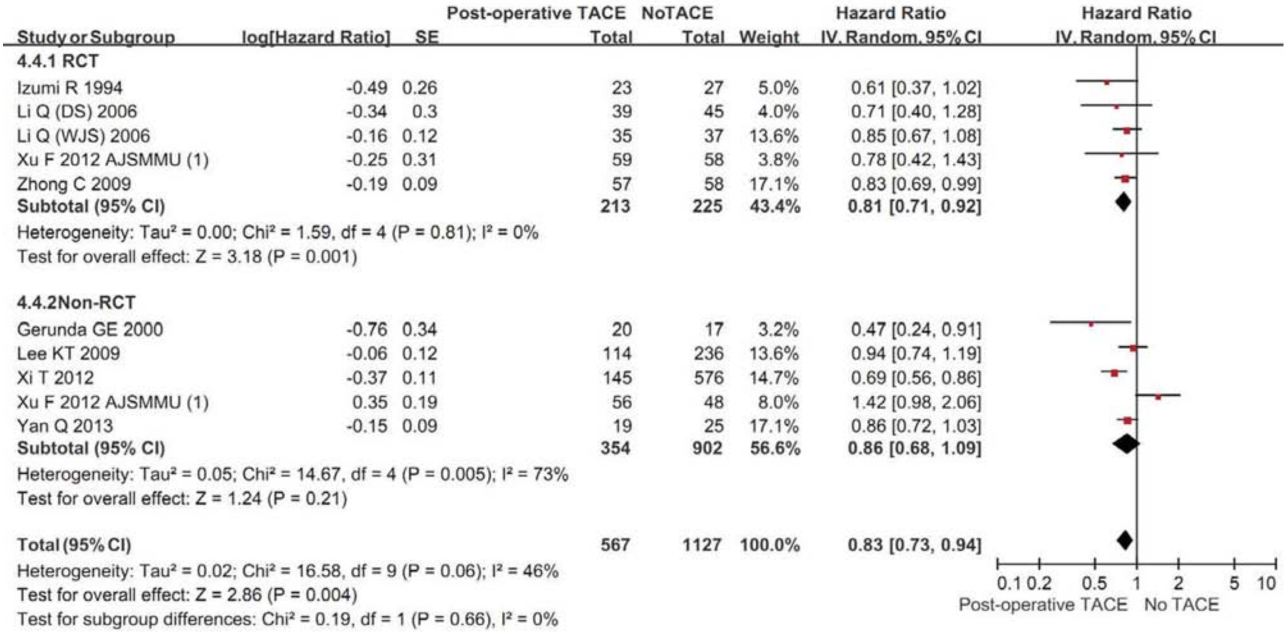

Supplementary Figure S18: Subgroup meta-analysis comparing the disease-free survival between hepatic resection with and without post-operative TACE groups according to the study design.

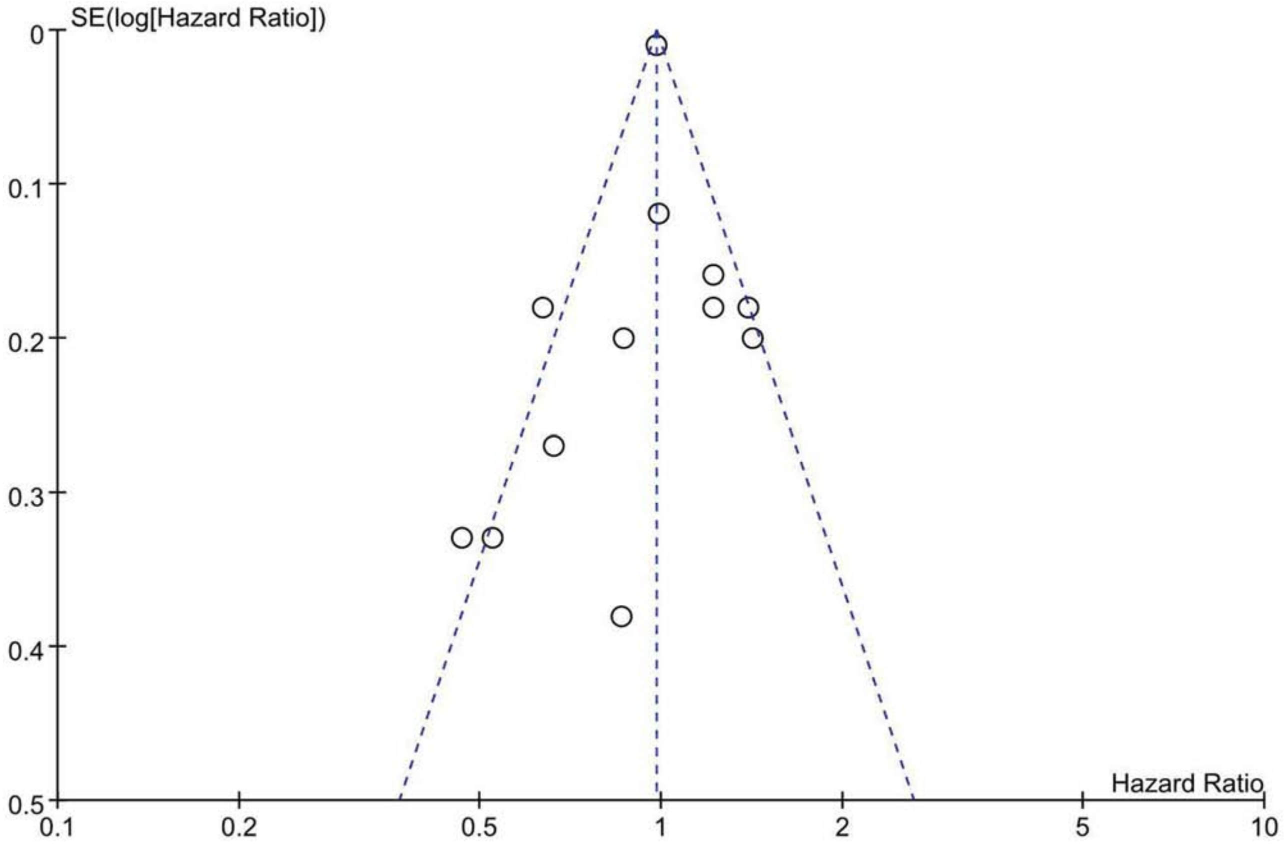

Supplementary Figure S19: Funnel plot regarding the comparison of rate free of recurrence between hepatic resection with and without post-operative TACE groups.

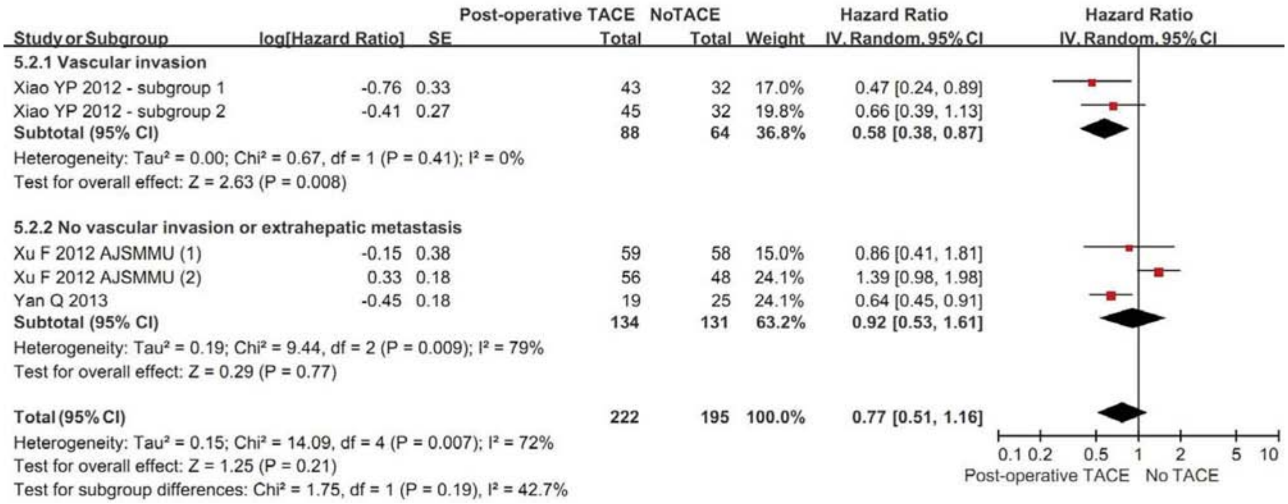

Supplementary Figure S20: Subgroup meta-analysis comparing the rate free of recurrence between hepatic resection with and without post-operative TACE groups according to the vascular invasion.

Study or Subgroup	log[Hazard Ratio]	SE	Post-operative TACE Total	NoTACE Total	Weight	Hazard Ratio IV, Random, 95% CI
**5.3.1 Large HCC**						
Chen XH 2010 - subgroup 3	0.35	0.2	166	228	13.8%	1.42 [0.96, 2.10]
Chen XH 2010 - subgroup 4	-0.14	0.2	135	126	13.8%	0.87 [0.59, 1.29]
Xiao YP 2012 - subgroup 1	-0.76	0.33	43	32	8.9%	0.47 [0.24, 0.89]
Xiao YP 2012 - subgroup 2	-0.41	0.27	45	32	10.9%	0.66 [0.39, 1.13]
**Subtotal (95% CI)**			**389**	**418**	**47.5%**	**0.82 [0.53, 1.28]**
Heterogeneity: Tau² = 0.14; Chi² = 10.42, df = 3 (P = 0.02); I² = 71%						
Test for overall effect: Z = 0.85 (P = 0.39)						
**5.3.2 Small HCC**						
Chen XH 2010 - subgroup 1	0.2	0.16	286	716	15.6%	1.22 [0.89, 1.67]
Chen XH 2010 - subgroup 2	0.2	0.18	190	234	14.7%	1.22 [0.86, 1.74]
Xu F 2012 AJSMMU (1)	-0.15	0.38	59	58	7.5%	0.86 [0.41, 1.81]
Yan Q 2013	-0.45	0.18	19	25	14.7%	0.64 [0.45, 0.91]
**Subtotal (95% CI)**			**554**	**1033**	**52.5%**	**0.97 [0.68, 1.38]**
Heterogeneity: Tau² = 0.08; Chi² = 9.19, df = 3 (P = 0.03); I² = 67%						
Test for overall effect: Z = 0.18 (P = 0.86)						
**Total (95% CI)**			**943**	**1451**	**100.0%**	**0.90 [0.70, 1.17]**
Heterogeneity: Tau² = 0.08; Chi² = 19.98, df = 7 (P = 0.006); I² = 65%						
Test for overall effect: Z = 0.76 (P = 0.45)						
Test for subgroup differences: Chi² = 0.31, df = 1 (P = 0.58), I² = 0%						

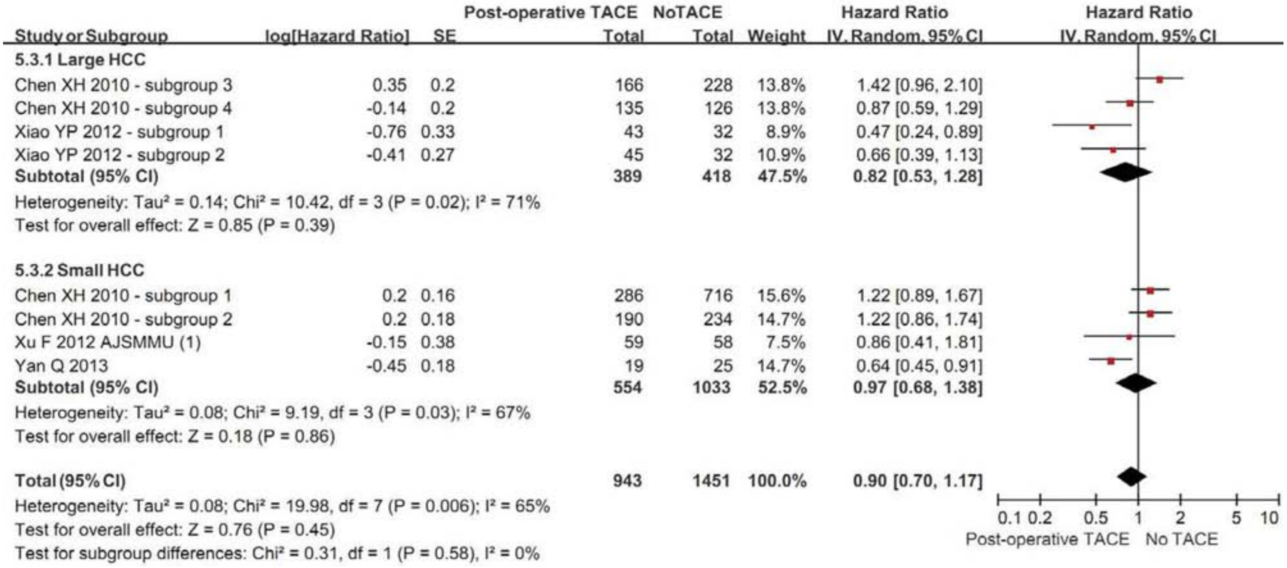

Supplementary Figure S21: Subgroup meta-analysis comparing the rate free of recurrence between hepatic resection with and without post-operative TACE groups according to the tumor size.

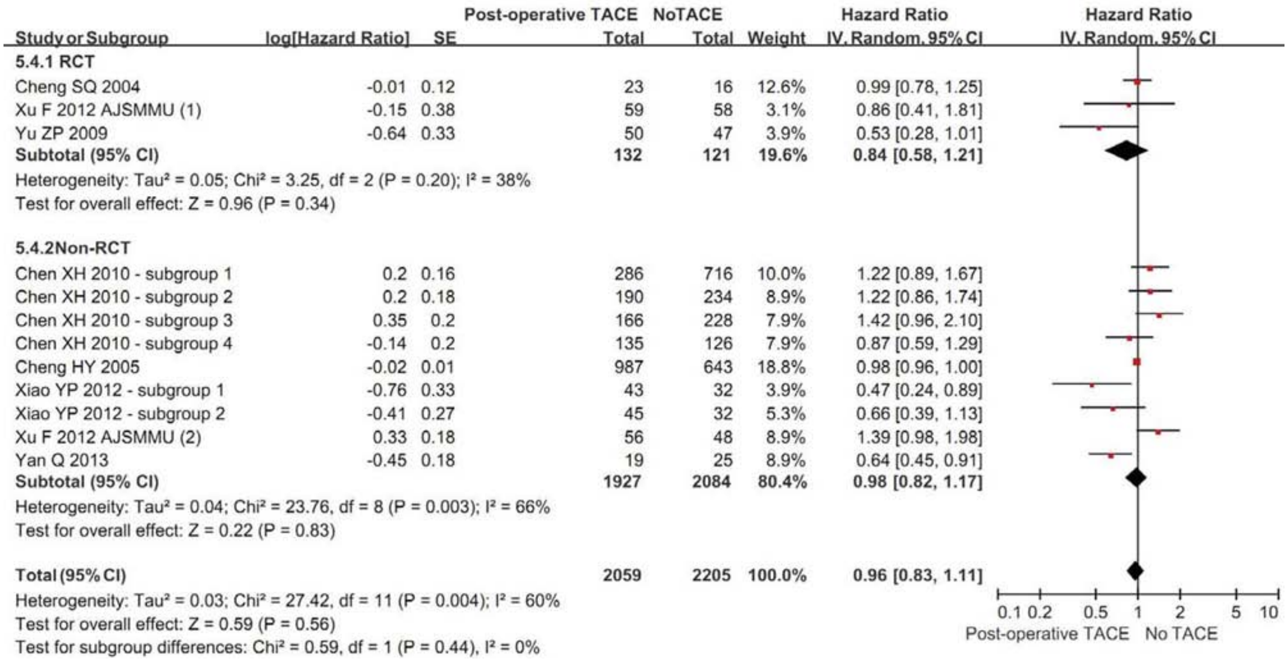

Supplementary Figure S22: Subgroup meta-analysis comparing the rate free of recurrence between hepatic resection with and without post-operative TACE groups according to the study design.

Supplementary Table S1: Quality assessment of non-randomized studies using NEWCASTLE - OTTAWA QUALITY ASSESSMENT SCALE COHORT STUDIES

First Author, Journal (Year)	Q1	Q2	Q3	Q4	Q5	Q6	Q7	Q8	Total score
**Adachi E, Cancer (1993)**	1 point	1 point	0 point	0 point	1 point	0 point	1 point	0 point	4 points
**Chen XH, Zhonghua Yi Xue Za Zhi (2010)**	1 point	1 point	0 point	0 point	2 points	1 point	0 point	1 point	4 points
**Chen XP, Dig Surg (2007)**	0 point	0 point	0 point	1 point	2 points	0 point	1 point	0 point	5 points
**Cheng HY, Zhonghua Zhong Liu Za Zhi (2005)**	1 point	1 point	0 point	0 point	0 point	0 point	0 point	0 point	2 points
**Cheng SQ, Zhonghua Zhong Liu Za Zhi (2005)**	1 point	1 point	0 point	1 point	2 points	0 point	1 point	1 point	7 points
**Choi GH, World J Surg (2007)**	1 point	1 point	0 point	1 point	2 points	1 point	1 point	1 point	8 points
**Di Carlo V, Hepato-gastroenterology (1998)**	0 point	0 point	0 point	0 point	2 points	0 point	1 point	0 point	3 points
**Gerunda GE, Liver Transpl (2000)**	1 point	1 point	0 point	0 point	2 points	1 point	1 point	0 point	6 points
**Hanazaki K, J Am Coll Surg (2000)**	1 point	1 point	0 point	0 point	0 point	1 point	1 point	0 point	4 points
**Harada T, Ann Surg (1996)**	1 point	1 point	0 point	0 point	2 points	1 point	1 point	0 point	6 points
**Jianyong L, Ann Hepatol (2014)**	1 point	1 point	0 point	0 point	2 points	1 point	1 point	0 point	6 points
**Kaibori M, Anticancer Research (2006)**	1 point	1 point	0 point	0 point	2 points	1 point	1 point	0 point	6 points
**Kim IS, Aliment Pharmacol Ther (2008)**	1 point	1 point	0 point	0 point	2 points	1 point	1 point	1 point	7 points
**Kishi Y, Hepato-gastroenterology (2012)**	1 point	1 point	0 point	0 point	1 point	1 point	1 point	0 point	5 points
**Lee KT, J Surg Oncol (2009)**	1 point	1 point	0 point	0 point	2 points	1 point	1 point	0 point	6 points
**Li F, Ir J Med Sci (2014)**	1 point	1 point	0 point	0 point	2 points	0 point	1 point	0 point	5 points

(*Continued*)

First Author, Journal (Year)	Q1	Q2	Q3	Q4	Q5	Q6	Q7	Q8	Total score
**Li KW, Hepato-gastroenterology (2012)**	1 point	1 point	0 point	0 point	2 points	1 point	1 point	0 point	6 points
**Liu YJ, Zhonghua Fang She Xue Za Zhi (2010)**	1 point	1 point	0 point	0 point	2 points	0 point	1 point	0 point	5 points
**Lu CD, World J Surg (1999)**	1 point	1 point	0 point	0 point	2 points	0 point	1 point	0 point	5 points
**Majno PE, Ann Surg (1997)**	1 point	1 point	0 point	0 point	0 point	0 point	1 point	0 point	3 points
**Nagasue N, Surgery (1989)**	1 point	1 point	0 point	0 point	1 point	0 point	1 point	0 point	4 points
**Nishikawa H, Int J Oncol (2013)**	1 point	1 point	0 point	0 point	2 points	1 point	1 point	0 point	6 points
**Ochiai T, Hepato-gastroenterology (2003)**	1 point	1 point	0 point	0 point	2 points	1 point	1 point	0 point	6 points
**Park JH, Cardiovasc Intervent Radiol (1993)**	1 point	1 point	0 point	0 point	0 point	1 point	1 point	0 point	4 points
**Sasaki A, Eur J Surg Oncol (2006)**	1 point	1 point	0 point	0 point	0 point	1 point	1 point	0 point	4 points
**Sugo H, World J Surg (2003)**	1 point	1 point	0 point	0 point	1 point	1 point	1 point	0 point	5 points
**Uchida M, World J Surg (1996)**	1 point	1 point	0 point	0 point	2 points	0 point	1 point	0 point	5 points
**Wang TH, Chinese J Cancer Prevention and Treatment (2010)**	1 point	1 point	0 point	0 point	2 points	0 point	1 point	0 point	5 points
**Wang QX, Zhonghua Wai Ke Za Zhi (2009)**	1 point	1 point	0 point	0 point	2 points	1 point	1 point	0 point	6 points
**Xi T, Hepato-gastroenterology (2012)**	1 point	1 point	0 point	0 point	2 points	1 point	1 point	0 point	6 points
**Xiao EH, Zhonghua Zhong Liu Za Zhi (2005)**	0 point	0 point	0 point	0 point	0 point	0 point	1 point	0 point	1 point
**Xiao YP, World Chinese J Digestology (2012)**	1 point	1 point	0 point	0 point	2 points	1 point	1 point	0 point	6 points

(*Continued*)

First Author, Journal (Year)	Q1	Q2	Q3	Q4	Q5	Q6	Q7	Q8	Total score
**Xu F, Academic J Second Military Medical University (2012) 2**	0 point	0 point	0 point	1 point	1 point	1 point	1 point	1 point	5 points
**Yan Q, Chin Med J (2013)**	1 point	1 point	0 point	0 point	2 points	1 point	1 point	0 point	6 points
**Yanaga K, HPB (2014)**	0 point	0 point	0 point	0 point	0 point	0 point	0 point	0 point	0 point
**Yang PS, Liver Transpl (2010)**	0 point	0 point	0 point	0 point	0 point	0 point	1 point	0 point	1 point
**Zhang Z, Cancer (2000)**	1 point	1 point	0 point	0 point	0 point	0 point	1 point	0 point	3 points

Supplementary Table S2: Quality assessment of non-randomized studies using NEWCASTLE - OTTAWA QUALITY ASSESSMENT SCALE CASE CONTROL STUDIES

First Author, Journal (Year)	Q1	Q2	Q3	Q4	Q5	Q6	Q7	Q8	Total score
**Kang JY, Korean J Hepatol (2010)**	0 point	0 point	0 point	1 point	2 points	1 point	1 point	0 point	5 points
**Paye F, Arch Surg (1998)**	0 point	1 point	0 point	1 point	2 points	1 point	1 point	0 point	6 points
**Ren ZG, World J Gastroenterol (2004)**	0 point	0 point	0 point	1 point	1 point	1 point	1 point	0 point	4 points
**Shi HY, J Surg Oncol (2014)**	1 point	1 point	1 point	1 point	0 point	0 point	0 point	0 point	4 points

Supplementary Table S3: Quality assessment of randomized studies using the Cochrane Collaboration's risk of bias tool

First Author, Journal (Year)	Cheng SQ, Zhonghua Zhong Liu Za Zhi (2004)	
**Entry**	**Judgment**	**Support for judgment**
**Random sequence generation (selection bias)**	High risk.	Quote: "57 patients with HCC were randomly divided into three groups according to the order of hospitalization".
**Allocation concealment (selection bias)**	Unclear risk.	Not described.
**Blinding of participants and personnel (performance bias)**	Unclear risk.	Not described.
**Blinding of outcome assessment (detection bias)**	Unclear risk.	Not described.
**Incomplete outcome data addressed (attrition bias)**	Unclear risk.	Not described.
**Selective reporting (reporting bias)**	Low risk.	Both recurrence and survival were reported. Review authors do not believe that bias will be introduced.
**First Author, Journal (Year)**	**Izumi R, Hepatology (1994)**	
**Entry**	**Judgment**	**Support for judgment**
**Random sequence generation (selection bias)**	Unclear risk.	Not described.
**Allocation concealment (selection bias)**	Unclear risk.	Not described.
**Blinding of participants and personnel (performance bias)**	Unclear risk.	Not described.
**Blinding of outcome assessment (detection bias)**	Unclear risk.	Not described.
**Incomplete outcome data addressed (attrition bias)**	Unclear risk.	Not described.
**Selective reporting (reporting bias)**	Low risk.	Both DFS and survival were reported. Review authors do not believe that bias will be introduced.
**First Author, Journal (Year)**	**Kaibori M, Dig Dis Sci (2012)**	
**Entry**	**Judgment**	**Support for judgment**
**Random sequence generation (selection bias)**	Unclear risk.	Quote: "They would be randomly selected for one of the above three groups". Comment: The authors did not mention the detailed methods for radnom sequence generation.
**Allocation concealment (selection bias)**	Low risk.	Quote: "They were randomized by the envelope method".
**Blinding of participants and personnel (performance bias)**	Unclear risk.	Not described.
**Blinding of outcome assessment (detection bias)**	Unclear risk.	Not described.
**Incomplete outcome data addressed (attrition bias)**	Unclear risk.	Not described.
**Selective reporting (reporting bias)**	Low risk.	Both recurrence and survival were reported. Review authors do not believe that bias will be introduced.

(*Continued*)

First Author, Journal (Year)	Li JQ, J Cancer Res Clin Oncol (1995)	
**Entry**	**Judgment**	**Support for judgment**
**Random sequence generation (selection bias)**	Unclear risk.	Quote: "140 patients were recruited to a randomized study". Comment: The authors did not mention the detailed methods for radnom sequence generation.
**Allocation concealment (selection bias)**	Unclear risk.	Not described.
**Blinding of participants and personnel (performance bias)**	Unclear risk.	Not described.
**Blinding of outcome assessment (detection bias)**	Unclear risk.	Not described.
**Incomplete outcome data addressed (attrition bias)**	Unclear risk.	Not described.
**Selective reporting (reporting bias)**	Low risk.	Both recurrence and survival were reported. Review authors do not believe that bias will be introduced.

First Author, Journal (Year)	Li Q, Dig surg (2006)	
**Entry**	**Judgment**	**Support for judgment**
**Random sequence generation (selection bias)**	Low risk.	Quote: "Random drawing of lots".
**Allocation concealment (selection bias)**	Unclear risk.	Not described.
**Blinding of participants and personnel (performance bias)**	High risk.	Quote: "a single-blind method".Comment: Review author did not recognize the detailed information regarding blinding of participants and personnel.
**Blinding of outcome assessment (detection bias)**	Unclear risk.	Not described.
**Incomplete outcome data addressed (attrition bias)**	Unclear risk.	Not described.
**Selective reporting (reporting bias)**	Low risk.	DFS was estimated according to the Methods. DFS was clearly reported in the Results. Review authors do not believe that bias will be introduced.

First Author, Journal (Year)	Li Q, World J Surg (2006)	
**Entry**	**Judgment**	**Support for judgment**
**Random sequence generation (selection bias)**	Low risk.	Quote: "The study cohort consisted of 112 patients with HCC and PVTT randomly divided into three groups". "The random drawing of lots".
**Allocation concealment (selection bias)**	Unclear risk.	Not described.
**Blinding of participants and personnel (performance bias)**	High risk.	Quote: "a single-blind method".Comment: Review author did not recognize the detailed information regarding blinding of participants and personnel.
**Blinding of outcome assessment (detection bias)**	Unclear risk.	Not described.

(*Continued*)

**Incomplete outcome data addressed (attrition bias)**	Unclear risk.	Not described.
**Selective reporting (reporting bias)**	Low risk.	DFS was estimated according to the Methods. DFS was clearly reported in the Results. Review authors do not believe that bias will be introduced.
**First Author, Journal (Year)**	**Peng BG, Am J Surg (2009)**	
**Entry**	**Judgment**	**Support for judgment**
**Random sequence generation (selection bias)**	Low risk.	Quote: "All patients were randomly assigned intothe control group and the TACE group". "computer-generated random numbers".
**Allocation concealment (selection bias)**	Low risk.	Quote: "Randomization was performed by means of sealed opaque envelopes containing computer-generated random numbers".
**Blinding of participants and personnel (performance bias)**	Unclear risk.	Not described.
**Blinding of outcome assessment (detection bias)**	Unclear risk.	Not described.
**Incomplete outcome data addressed (attrition bias)**	Low risk.	Quote: "Twelve patients in the TACE group and 10 patients in the control group were lost during follow-up."Comments: Patients lost to follow-up balanced in numbers between groups, with similar reasons for missing data between groups.
**Selective reporting (reporting bias)**	High risk.	Quote: "When there was evidence of recurrence, enhanced computerized axial tomography (CAT) was employed to confirm the diagnosis". Comments: Desipte recurrence was mentioned in the Methods section, the recurrence data during follow-up were not reported.
**First Author, Journal (Year)**	**Tang QH, Academic J Second Military Medical University (2009)**	
**Entry**	**Judgment**	**Support for judgment**
**Random sequence generation (selection bias)**	Low risk.	Quote: "prospectively randomized into surgical resection group or preoperative TACE group" "computer-generated random numbers".
**Allocation concealment (selection bias)**	High risk.	Quote: "numbered according to the date of hospitalization".
**Blinding of participants and personnel (performance bias)**	High risk.	Quote: "patients, their relatives, nurses did not know the the detailed treatment".
**Blinding of outcome assessment (detection bias)**	Low risk.	Quote: "Data were analyzed by two statisticians independently". "Statisticians did not know the assignment of groups".
**Incomplete outcome data addressed (attrition bias)**	High risk.	Quote: "52 patients were assigned to TACE+surgery group, but 5 patients did not undergo surgery after TACE due to the extrahepatic metastasis (n = 4) and liver function deterioration (n = 1)." "56 patients were assigned to surgery group, all of them underwent surgery".
**Selective reporting (reporting bias)**	Low risk.	Both DFS and survival were reported. Review authors do not believe that bias will be introduced.

(*Continued*)

First Author, Journal (Year)		Wu CC, Br J Surg (1995)
**Entry**	**Judgment**	**Support for judgment**
**Random sequence generation (selection bias)**	Unclear risk.	Quote: "The 52 patients were randomized into two groups". Comment: The authors did not mention the detailed methods for radnom sequence generation.
**Allocation concealment (selection bias)**	Unclear risk.	Not described.
**Blinding of participants and personnel (performance bias)**	Unclear risk.	Not described.
**Blinding of outcome assessment (detection bias)**	Unclear risk.	Not described.
**Incomplete outcome data addressed (attrition bias)**	Unclear risk.	Not described.
**Selective reporting (reporting bias)**	Low risk.	Both DFS and survival were reported. Review authors do not believe that bias will be introduced.

First Author, Journal (Year)		Xu F, Academic J Second Military Medical University (2012)
**Entry**	**Judgment**	**Support for judgment**
**Random sequence generation (selection bias)**	Low risk.	Quote: "117 patients were randomly divided into 2 groups". "computer-generated random numbers".
**Allocation concealment (selection bias)**	High risk.	Quote: "a list of random numbers, in which the first 60 were assigned to the TACE+surgery group, and the other 60 were assigned to the TACE group".
**Blinding of participants and personnel (performance bias)**	Unclear risk.	Not described.
**Blinding of outcome assessment (detection bias)**	Unclear risk.	Not described.
**Incomplete outcome data addressed (attrition bias)**	Low risk.	Quote: "None of patients were lost to follow-up".
**Selective reporting (reporting bias)**	Low risk.	DFS was estimated according to the Methods. DFS was clearly reported in the Results. Review authors do not believe that bias will be introduced.

First Author, Journal (Year)		Yamasaki S, Jpn J Cancer Res (1996)
**Entry**	**Judgment**	**Support for judgment**
**Random sequence generation (selection bias)**	Unclear risk.	Not described.
**Allocation concealment (selection bias)**	Unclear risk.	Quote: "Patients were randomized using the envelop method".Comment: it remains unclear about whether or not envelop was sealed or opaque.
**Blinding of participants and personnel (performance bias)**	Unclear risk.	Not described.
**Blinding of outcome assessment (detection bias)**	Unclear risk.	Not described.

(*Continued*)

**Incomplete outcome data addressed (attrition bias)**	Unclear risk.	Not described.
**Selective reporting (reporting bias)**	Low risk.	Both cancer-free survival and survival rates were reported. Review authors do not believe that bias will be introduced.
**First Author, Journal (Year)**	**Yu ZP, J Pract Med (2009)**	
**Entry**	**Judgment**	**Support for judgment**
**Random sequence generation (selection bias)**	Unclear risk.	Not described.
**Allocation concealment (selection bias)**	Unclear risk.	Not described.
**Blinding of participants and personnel (performance bias)**	Unclear risk.	Not described.
**Blinding of outcome assessment (detection bias)**	Unclear risk.	Not described.
**Incomplete outcome data addressed (attrition bias)**	Low risk.	Quote: "all patients have been followed for more than 2 years".
**Selective reporting (reporting bias)**	Low risk.	Both intrahepatic recurrence and survival rates were reported. Review authors do not believe that bias will be introduced.
**First Author, Journal (Year)**	**Zhong C, J Cancer Res Clin Oncol (2009)**	
**Entry**	**Judgment**	**Support for judgment**
**Random sequence generation (selection bias)**	Unclear risk.	Quote: "A total of 118 patients were initially randomized to undergo partial hepatectomy and adjuvant TACE (HT arm) or partial hepatectomy alone (HA arm) by drawing consecutive sealed envelopes".Comment: The authors did not mention the detailed methods for radnom sequence generation.
**Allocation concealment (selection bias)**	Low risk.	Quote: "drawing consecutive sealed envelopes".
**Blinding of participants and personnel (performance bias)**	Unclear risk.	Not described.
**Blinding of outcome assessment (detection bias)**	Unclear risk.	Not described.
**Incomplete outcome data addressed (attrition bias)**	Low risk.	Quote: "1 patient in hepatectomy alone group was lost to follow-up".
**Selective reporting (reporting bias)**	Low risk.	Both DFS and survival were reported. Review authors do not believe that bias will be introduced.
**First Author, Journal (Year)**	**Zhou WP, Ann Surg (2009)**	
**Entry**	**Judgment**	**Support for judgment**
**Random sequence generation (selection bias)**	Unclear risk.	Quote: "All eligible patients were randomly assigned to either the preoperative TACE group or the control group by drawing sealed, consecutively numbered, and opaque envelopes after completing the preoperative evaluation". Comment: The authors did not mention the detailed methods for radnom sequence generation.

(*Continued*)

**Allocation concealment (selection bias)**	Low risk.	Quote: "drawing sealed, consecutively numbered, and opaque envelopes".
**Blinding of participants and personnel (performance bias)**	Unclear risk.	Not described.
**Blinding of outcome assessment (detection bias)**	Unclear risk.	Not described.
**Incomplete outcome data addressed (attrition bias)**	Low risk.	Quote: "Five patients were lost to follow-up after discharge from hospital". "A total of 108 patients were left for final analysis"
**Selective reporting (reporting bias)**	Low risk.	Both DFS and survival were reported. Review authors do not believe that bias will be introduced.
